# Predictors of Long COVID: older adults, women, and an incomplete COVID-19 vaccination schedule^*^

**DOI:** 10.1590/1518-8345.7954.4816

**Published:** 2026-07-20

**Authors:** Karina Marques Prediger, Ana Cristina Ribeiro La Scaléa, Silvia Carla da Silva André Uehara

**Affiliations:** 1 Universidade Federal de São Carlos, Departamento de Enfermagem, São Carlos, SP, Brazil.; 2 Scholarship holder at the Fundação de Amparo à Pesquisa do Estado de São Paulo (FAPESP), Brazil.; 3 Scholarship holder at the Coordenação de Aperfeiçoamento de Pessoal de Nível Superior (CAPES), Brazil.

**Keywords:** Post-Acute COVID-19 Syndrome, Prevalence, Female, Age Factors, Vaccines, Signs and Symptoms

## Abstract

**Objective:**

to analyze the symptoms of long COVID in patients treated in Primary Health Care, considering gender, age, and vaccination status.

**Method:**

this is a cross-sectional and analytical study conducted with a sample of 350 patients with long COVID treated in Primary Health Care services. Data were collected through the analysis of medical records and analyzed using the Poisson regression model.

**Results:**

older adults had an 88% higher prevalence of shortness of breath as a symptom of long COVID; females had a 13.5 times higher prevalence of hair loss; and unvaccinated individuals had a 3.64 times higher prevalence of reporting chest pain.

**Conclusion:**

identifying the most prevalent symptoms of long COVID, taking into account age group, gender, and vaccination status, can contribute to clinical evaluation and the development of protocols to guide the appropriate management of these individuals.

## Highlights

**(1)** Older people had a higher prevalence of shortness of breath in LC. **(2)** Women had a higher prevalence of hair loss in LC. **(3)** Unvaccinated people had a higher prevalence of chest pain in LC

## Introduction

Long COVID is characterized by a history of COVID-19 infection, whose symptoms persist or fluctuate for at least two months after the acute phase and cannot be explained by an alternative diagnosis. Among the most frequent symptoms of long COVID are fatigue, shortness of breath, cognitive dysfunction, and others that impact the patient’s daily activities^1^.

The global prevalence of long COVID is estimated to be approximately 36%, varying by geographic region, with an estimated prevalence of 35% in Asia, 39% in Europe, 30% in North America, and 51% in South America^2^. In Brazil, there is also regional variation in the prevalence of long COVID. Between March 2020 and March 2023, the states with the highest rates per 100,000 inhabitants were Goiás, the Federal District, Paraná, Rio Grande do Sul, Tocantins, and São Paulo^3^.

From September 2021 to December 2024, 167,138 individual visits were recorded in Primary Health Care (PHC) attributed to an unspecified post-COVID-19 health condition (ICD-U09.9). The state of São Paulo had the highest cumulative incidence of post-COVID care in the country, with 2,026.9 visits per 100,000 inhabitants, followed by the states of Amazonas, Rio Grande do Sul, Minas Gerais, and Tocantins^3^.

Thus, although the coefficients of long COVID vary globally and nationally, it is possible to observe that a portion of patients infected with COVID-19 may develop prolonged symptoms^4^. Long COVID is a condition that encompasses heterogeneous and multisystemic manifestations, involving different organ systems, such as cardiovascular, gastrointestinal, endocrine, and neuropsychiatric^5^ ^-^ ^7^. Therefore, healthcare professionals who treat patients with these symptoms may easily attribute them to psychosomatic causes, such as depression and anxiety, which can hinder the diagnosis of long COVID^4^.

It should be noted that patients who developed severe COVID-19 tend to experience prolonged symptoms after the acute phase of the disease. In this context, the prevalence of persistent symptoms was verified in outpatients, with 5% to 26% of these individuals presenting prolonged symptoms of COVID-19 for a period of one to three months and 2% to 62% presenting symptoms for a period of three to six months after diagnosis of the acute disease^8^.

The pathophysiology of long COVID may be related to different mechanisms, depending on the organ system affected. Thus, the development of long COVID involves ongoing sequelae of severely damaged tissues, the persistence of SARS-CoV-2 in organs, dysregulation of the immune system that triggers autoimmunity and chronic inflammation, tissue hypoxia, and endothelial damage^5^.

Although the literature describes the symptoms manifested in long COVID^1^ ^,^ ^4^, given the similarity to other diseases, characterizing the most prevalent symptoms according to gender, age group, and COVID-19 vaccination status can help identify the population group most likely to develop this condition. Thus, this study aims to analyze the symptoms of long COVID in patients assisted in Primary Health Care, considering gender, age, and vaccination status.

## Method

### Type of study

This is a cross-sectional, analytical study designed in accordance with the Strengthening the Reporting of Observational Studies in Epidemiology (STROBE)^9^ guidelines. 

### Scenario

This study was conducted in the municipality of São Carlos, located in the state of São Paulo. The data were obtained from the Health Surveillance Services and the Department of Management and Outpatient Care, the sector responsible for Primary Health Care (PHC) services.

### Period

For the sample calculation, data on COVID-19 cases in the adult population reported between February 26, 2020, and February 26, 2024, were used. Thus, it was possible to identify, in this interval, the ICD-Z20 diagnosis (confirmed COVID-19) and at least one return to PHC services two months after the date of acute COVID-19 infection. Data collection took place between August 2023 and June 2024.

### Study population and sample

A sample calculation was performed considering all cases of COVID-19 diagnosed in the population over 18 years of age in the municipality, covering the period from February 26, 2020, to February 26, 2024, using either Rapid Testing (RT) or Reverse Transcription Polymerase Chain Reaction (RT-PCR). In the city of São Carlos, SP, 62,977 positive cases of COVID-19 were reported in individuals aged 18 years or older during the study’s analysis period. The choice of prevalence was based on data from the literature^10^, which indicates that approximately 50% of COVID-19 cases present symptoms of long COVID.

For the sample calculation, a significance level of 5% (α = 0.05), a tolerable absolute error of 5 percentage points (d = 0.05), and an estimated finite population of N = 31,489 patients with long COVID over the age of 18 in the municipality of interest were assumed. The expected prevalence used was p = 0.356, corresponding to the frequency of symptom fatigue, one of the most reported manifestations in previous studies on long COVID. Thus, the sample size adopted in this study was n = 350. The calculation was performed using the following equation:

$$n=\frac{z_{\left(1-\frac{\alpha }{2}\right)}^{2}Np\left(1-p\right)}{d^{2}\left(N-1\right)+z_{\left(1-\frac{\alpha }{2}\right)}^{2}p\left(1-p\right)}$$

Where *p* is the expected prevalence, *d* is the tolerable absolute error, *N* is the population size, and *z* is the value of the standard normal distribution corresponding to the desired confidence level. 

### Selection criteria

Adult patients over 18 years of age who tested positive for COVID-19 and returned to PHC services with symptoms of long COVID, as defined by the WHO, which describes the symptoms as recurrent or fluctuating for a minimum period of two months after infection with the acute disease, were included in the study^1^. Patients whose medical records were inaccessible, who did not reside in locations affiliated with PHC, or who had died were excluded from the study.

### Study variables

The study variables were gender, age, race/color, comorbidities, vaccination schedule, and reported symptoms. Reported symptoms included changes in menstruation, anxiety, fatigue, runny nose, diarrhea, difficulty concentrating, abdominal pain, joint pain, headache, sore throat, lower limb pain, upper limb pain, muscle pain, back/lumbar pain, ear pain, chest pain, shortness of breath, fever, tingling in the lower and/or upper limbs, weakness/loss of muscle strength, insomnia, nausea, odynophagia, memory loss/confusion, loss of smell, loss of taste, hair loss, dental complaints of tooth loss, dizziness, cough, sadness/depression, and vomiting. This study considered self-reported symptoms compatible with the WHO^1^ definition of long COVID, meaning symptoms that usually start three months after the acute phase of infection, last at least two months, and have no other identifiable clinical explanation.

### Instruments used for data collection

For data collection, spreadsheets were created in Microsoft Excel containing the variables of interest. Initially, separate tabs were designed to collect data from the various databases used in this research, which were obtained from SisCOVID (an acronym in Portuguese), the COVID-19 notification system of the municipality of São Carlos, SP. In the next tab, data on vaccination status were entered, obtained from VaciVida, a system developed by the State of São Paulo. Finally, in the last tab, data on long COVID symptoms obtained from the Electronic Citizen Record (PEC, acronym in Portuguese) were entered. Measures were taken to minimize bias, including training data collectors - two undergraduates, two master’s students, and one doctoral student in Health Science - using predefined inclusion and exclusion criteria, and standardizing data collection using a structured, digitized spreadsheet. In addition, the choice of an appropriate significance level (α = 0.05), adjustment for multiple comparisons, the application of suitable statistical methods, and a robust sample size are all noteworthy considerations.

### Data collection

First, confirmed cases of COVID-19 were identified in SisCOVID; subsequently, the PEC was consulted to determine whether patients infected with SARS-CoV-2 returned to PHC with reports of long COVID symptoms, as defined by the WHO^1^. Through SisCOVID, it was also possible to obtain information on consultations by users with complaints of respiratory symptoms after the acute phase of the disease; through PEC, the symptoms of long COVID reported by users, which affected different organ systems of the bod, were verified. In addition, data on vaccination were obtained from the VaciVida website, together with municipal epidemiological surveillance.

### Data processing and analysis

Initially, the data were described using absolute frequencies and percentages (for qualitative variables) and measures of centrality and dispersion, such as the mean, standard deviation, minimum, median, and maximum (for quantitative variables). To estimate the crude prevalence ratio of long COVID symptoms in relation to the variables of interest, a Poisson regression model with robust variance was used^11^. In analyses where the frequency of symptoms was zero, Fisher’s exact test was performed. The use of Missing Data was indicated in the footnotes of the tables to ensure the transparency of the data presented, maintaining the number of missing cases as observed, without imputation. All analyses were performed using SAS 9.4 software^12^, adopting a significance level of 5%.

### Ethical aspects

In accordance with Resolution No. 510/2016, the project was approved by the Research Ethics Committee of the Federal University of São Carlos (CAAE: 67128023.4.0000.5504; CAAE: 74911623.5.0000.5504).

## Results

A total of 3,778 reported and confirmed cases of COVID-19 were analyzed to obtain a sample of 350 cases of long COVID, corresponding to 12% of acute cases in the population aged 18 years and older in the municipality of São Carlos, São Paulo. The study sample consists of 67.1% (235) females and 32.9% (115) males; 61.6% (215) declared themselves to be white, 30.1% (105) brown, 8.0% (28) black, 0.3% (1) yellow, and 1 case had no race/color recorded. Regarding age group, 77.1% (270) were people under 60 years of age, and 22.9% (80) were people aged 60 years or older. The average age was 46.9 years (Table 1).

When comparing the symptoms of long COVID presented by patients, considering gender, it is noteworthy that females had a 13.5 times higher prevalence of hair loss compared to males (Table 2).

**Table 1:** Demographic and clinical characterization of the study sample. São Carlos, SP, Brazil, 2024

Characteristics	Frequency (n = 350)	Percentage (%)
**Gender**		
Female	235	67.1
Male	115	32.9
**Age**		
Mean	46.9 [15.8]	-
Median (Min* e Max^†^)	47.0 [14.0 e 88.0]	-
**Age group**		
< 60 years	270	77.1%
≥ 60 years	80	22.9%
**Race/ Color**		
Yellow	1	0.3
White	215	61.6
Brown	105	30.1
Black	28	8.0
Missing	1	-
**Comorbities**		
Hemoglobinopathies	1	0.3
Liver cirrhosis	0	0
Cardiovascular diseases	17	4.9
Neurological diseases	4	1.1
Diabetes Mellitus (Type1 and Type2)	53	15.1
Chronic Kidney Disease	4	1.1
Systemic Arterial Hypertension	106	30.3
Immunosuppressed	0	0
Obesity	56	16
Severe chronic pneumopathies	21	6.0
Others	51	14.6

*Min = Minimum; ^†^Max = Maximum

**Table 2:** Comparison of the presence of long COVID symptoms, considering the sex of the study population. São Carlos, SP, Brazil, 2024

Symptoms of long COVID	F* (n = 235)	M^†^ (n = 115)	Prevalence ratio (F* *vs* M^†^) [CI 95%]^‡^	p-value^§^
Changes in menstruation^||^	8 (3.6%)	0 (0%)	-	0.06^¶^
Anxiety^||^	14 (6.3%)	7 (6.7%)	0.94 (0.39; 2.25)	0.89
Fatigue	44 (18.7%)	13 (11.3%)	1.66 (0.93; 2.95)	0.09
Runny nose	32 (13.7%)	14 (12.2%)	1.12 (0.62; 2.02)	0.70
Diarrhea	4 (1.7%)	0 (0%)	-	0.31^¶^
Difficulty concentrating^||^	5 (2.2%)	0 (0%)	-	0.18^¶^
Abdominal pain^||^	13 (58%)	8 (7.6%)	0.76 (0.33; 1.78)	0.53
Joint pain^||^	4 (1.8%)	3 (2.9%)	0.63 (0.14; 2.74)	0.53
Headache	72 (30.6%)	25 (21.7%)	1.41 (0.95; 2.1)	0.09
Sore throat	46 (19.6%)	20 (17.4%)	1.13 (0.7; 1.81)	0.63
Pain in lower limbs^||^	24 (10.7%)	15 (14.3%)	0.75 (0.41; 1.37)	0.35
Pain in upper limbs^||^	9 (4.0%)	6 (5.7%)	0.7 (0.26; 1.92)	0.49
Muscle pain	37 (15.7%)	14 (12.2%)	1.29 (0.73; 2.29)	0.38
Back/lumbar pain^||^	22 (9.8%)	8 (7.6%)	1.29 (0.59; 2.8)	0.52
Ear pain^||^	1 (0.4%)	0 (0%)	-	0.99^¶^
Chest pain^||^	10 (4.5%)	7 (6.7)	0.67 (0.26; 1.71)	0.40
Chest pain^||^	10 (4.5%)	3 (2.9%)	1.56 (0.44; 5.56)	0.49
Shortness of breath	33 (14.0%)	23 (20.0%)	0.7 (0.43; 1.14)	0.15
Fever	26 (11.1%)	8 (7.0%)	1.59 (0.74; 3.4)	0.23
Tingling in lower limbs^||^	11 (4.9%)	5 (4.8%)	1.03 (0.37; 2.89)	0.95
Tingling in upper limbs^||^	10 (4.5%)	1 (1.0%)	4.69 (0.61; 36.14)	0.14
Weakness/loss of muscle strength^||^	21 (9.4%)	9 (8.6%)	1.09 (0.52; 2.31)	0.81
Insomnia^||^	15 (6.7%)	4 (3.8%)	1.76 (0.6; 5.17)	0.31
Nausea	7 (3.0%)	1 (0.9%)	3.43 (0.43; 27.51)	0.25
Odynophagia^||^	1 (0.4%)	1 (1.0%)	0.47 (0.03; 7.42)	0.59
Memory loss/confusion^||^	10 (4.5%)	5 (4.8%)	0.94 (0.33; 2.67)	0.90
Loss of smell	3 (1.3%)	4 (3.5%)	0.37 (0.08; 1.61)	0.18
Loss of taste	3 (1.3%)	1 (0.9%)	1.47 (0.15; 13.96)	0.74
Hair loss^||^	29 (12.9%)	1 (1.0%)	13.59 (1.88; 98.45)	0.01
Dental complaints of tooth loss^||^	2 (0.9%)	1 (1.0%)	0.94 (0.09; 10.22)	0.96
Dizziness	11 (4.9%)	4 (3.8%)	1.29 (0.42; 3.95)	0.66
Cough	59 (25.1%)	27 (23.5%)	1.07 (0.72; 1.59)	0.74
Sadness/depression^||^	11 (4.9%)	4 (3.8%)	1.29 (0.42; 3.95)	0.66
Vomiting	9 (3.8%)	3 (2.6%)	1.47 (0.41; 5.32)	0.56

*F = Female; ^†^M = Male; ^‡^CI = Confidence Interval; ^§^P-value = Significance level; ^||^Missing 20; ^¶^Value referring to Fisher’s exact test

When comparing the symptoms of long COVID in patients, considering the age group, it is noteworthy that people aged 60 years or older had an 88% higher prevalence of shortness of breath, in addition to a 52% decrease in the prevalence of headache, 66% in sore throat, and 63% in muscle pain compared to younger adults (Table 3).

The results indicated that people with long COVID who had not received any doses of the COVID-19 vaccine had a 3.64 times higher prevalence of chest pain (Table 4).

The results show that 27.2% (96) of people with long COVID had received two doses of the COVID-19 vaccine, with headache being the most common symptom in this group, present in 31.3% (30) of the analyzed cases. It was found that, regardless of the number of doses administered against COVID-19, the common symptoms of long COVID among people were headache, sore throat, and cough (Table 5).

**Table 3:** Comparison of the presence of long COVID symptoms, considering the age group of the study population. São Carlos, SP, Brazil, 2024

Description of long COVID symptoms	< 60 years (n = 270)	≥ 60 years (n = 80)	Prevalence ratio (≥ 60 *vs* < 60) [CI 95%]*	p-value^†^
Changes in menstruation^‡^	8 (3.2%)	0 (0%)	-	0.21^§^
Anxiety^‡^	19 (7.6%)	2 (2.6%)	0.34 (0.08; 1.42)	0.14
Fatigue	47 (17.4%)	10 (12.5%)	0.72 (0.38; 1.36)	0.92
Runny nose	41 (15.2%)	5 (6.3%)	0.41 (0.17; 1)	0.05
Diarrhea	3 (1.1%)	1 (1.3%)	1.13 (0.12; 10.67)	0.92
Difficulty concentrating^‡^	5 (2.0%)	0 (0%)	-	0.60^§^
Abdominal pain^‡^	16 (6.4%)	5 (6.4%)	1.01 (0.38; 2.66)	0.99
Joint pain^‡^	5 (2.0%)	2 (2.6%)	1.29 (0.25; 6.5)	0.76
Headache	85 (31.5%)	12 (15.0%)	0.48 (0.27; 0.83)	<0.01
Sore throat	60 (22.2%)	6 (7.5%)	0.34 (0.15; 0.89)	0.03
Pain in lower limbs^‡^	29 (11.6%)	10 (12.8%)	1.11 (0.57; 2.17)	0.76
Pain in upper limbs^‡^	11 (4.4%)	4 (5.1%)	1.17 (0.38; 3.57)	0.78
Muscle pain	46 (17.0%)	5 (6.3%)	0.37 (0.15; 0.89)	0.03
Back/lower back pain^‡^	23 (9.2%)	7 (9.0%)	0.98 (0.44; 2.19)	0.96
Ear pain^‡^	0 (0%)	1 (1.3%)	-	0.24^§^
Chest pain^‡^	15 (6.0%)	2 (2.6%)	0.43 (0.1; 1.84)	0.25
Chest discomfort^‡^	12 (4.8%)	1 (1.3%)	0.27 (0.04; 2.03)	0.20
Shortness of breath	36 (13.3%)	20 (25.0%)	1.88 (1.15; 3.05)	0.01
Tingling/throbbing in lower limbs^‡^	14 (5.6%)	2 (2.6%)	0.46 (0.11; 1.98)	0.30
Tingling/throbbing in upper limbs^‡^	8 (3.2%)	3 (3.8%)	1.21 (0.33; 4.44)	0.78
Weakness/loss of muscle strength^‡^	20 (8.0%)	10 (12.8%)	1.61 (0.79; 3.29)	0.19
Insomnia^‡^	15 (6.0%)	4 (5.1%)	0.86 (0.29; 2.51)	0.78
Fever	28 (10.4%)	6 (7.5%)	0.72 (0.31; 1.68)	0.45
Nausea	8 (3.0%)	0 (0%)	-	0.21
Odynophagia^‡^	2 (0.8%)	0 (0%)	-	0.99^§^
Memory loss/confusion^‡^	10 (4.0%)	5 (6.4%)	1.61 (0.57; 4.57)	0.37
Loss of smell	5 (1.9%)	2 (2.55)	1.35 (0.27; 6.83)	0.72
Loss of taste	3 (1.1%)	1 (1.3%)	1.13 (0.12; 10.67)	0.92
Hair loss^‡^	27 (10.8%)	3 (3.8%)	0.36 (011; 1.15)	0.08
Dental complaint of tooth loss after COVID-19	2 (0.8%)	1 (1.3%)	1.61 (0.15; 17.51)	0.70
Sadness/depression	11 (4.4%)	4 (5.1%)	1.17 (0.38; 3.57)	0.78
Dizziness	11 (4.4%)	4 (5.1%)	1.17 (0.38; 3.57)	0.78
Cough	73 (27.0%)	13 (16.3%)	0.6 (0.35; 1.03)	0.06
Vomiting	10 (3.7%)	2 (2.5%)	0.68 (0.15; 3.02)	0.61

*CI = Confidence Interval; ^†^P-value = Significance level; ^‡^Missing 21; ^§^Value referring to Fisher’s exact test

**Table 4:** Comparison of the presence of long COVID symptoms, considering the vaccination schedule of the study population. São Carlos, SP, Brazil, 2024

Description of long COVID symptoms	Before the vaccine (n = 295)	After the vaccine (n = 47)	Prevalence ratio [CI 95%]*	p-value^†^
Changes in menstruation^‡^	6 (2.1%)	2 (4.8%)	2.22 (0.46; 10.65)	0.32
Anxiety^‡^	18 (6.4%)	3 (7.1%)	1.11 (0.34; 3.61)	0.86
Fatigue	53 (18.0%)	4 (8.5%)	0.47 (0.18; 1.25)	0.13
Runny nose	36 (12.2%)	10 (21.3%)	1.74 (0.93; 3.26)	0.09
Diarrhea	4 (1.4%)	0 (0%)	-	0.99^§^
Difficulty concentrating^‡^	4 (1.4%)	1 (2.4%)	1.67 (0.19; 14.56)	0.64
Abdominal pain^‡^	19 (6.8%)	2 (4.8%)	0.7 (0.17; 2.9)	0.63
Joint pain^‡^	6 (2.1%)	1 (2.4%)	1.11 (0.14; 9)	0.92
Headache	83 (28.1%)	13 (27.7%)	0.98 (0.6; 1.62)	0.95
Sore throat	53 (18.0%)	12 (25.5%)	1.42 (0.82; 2.45)	0.21
Pain in lower limbs^‡^	36 (12.9%)	2 (4.8%)	0.37 (0.09; 1.48)	0.16
Pain in upper limbs^‡^	14 (5.0%)	1 (2.4%)	0.48 (0.06; 3.53)	0.47
Muscle pain	45 (15.3%)	6 (12.8%)	0.84 (0.38; 1.85)	0.66
Back/lumbar pain^‡^	26 (9.3%)	3 (7.1%)	0.77 (0.24; 2.43)	0.65
Ear pain^‡^	1 (0.4%)	0 (0%)	-	0.99^§^
Chest pain	11 (3.9%)	6 (14.3%)	3.64 (1.42; 9.31)	<0.01
Chest pain^‡^	10 (3.6%)	3 (7.1%)	2 (0.57; 6.97)	0.28
Shortness of breath	48 (16.3%)	7 (14.9%)	0.92 (0.44; 1.9)	0.81
Tingling/throbbing in lower limbs^‡^	11 (3.9%)	4 (9.5%)	2.42 (0.81; 7.26)	0.11
Tingling/throbbing in upper limbs^‡^	10 (3.6%)	1 (2.4%)	0.67 (0.09; 5.08)	0.70
Weakness/loss of muscle strength^‡^	27 (9.6%)	3 (7.1%)	0.74 (0.24; 2.33)	0.61
Insomnia^‡^	15 (5.4%)	3 (7.1%)	1.33 (0.4; 4.41)	0.64
Fever	30 (10.2%)	4 (8.5%)	0.84 (0.31; 2.27)	0.73
Nausea	7 (2.4%)	1 (2.1%)	0.9 (0.11; 7.12)	0.92
Odynophagia^‡^	1 (0.4%)	1 (2.4%)	6.67 (0.43; 104.57)	0.18
Memory loss/confusion^‡^	13 (4.6%)	1 (2.4%)	0.51 (0.07; 3.82)	0.51
Loss of smell	5 (1.7%)	2 (4.3%)	2.51 (0.5; 12.57)	0.26
Loss of taste	3 (1.0%)	1 (2.1%)	2.09 (0.22; 19.69)	0.52
Hair loss^‡^	26 (9.3%)	4 (9.5%)	1.03 (0.38; 2.79)	0.96
Dental complaint of post-COVID tooth loss^‡^	3 (1.11%)	0 (0%)	-	0.99^§^
Sadness/depression^‡^	12 (4.3%)	2 (4.8%)	1.11 (0.26; 4.79)	0.89
Dizziness^‡^	12 (4.3%)	3 (7.1%)	1.67 (0.49; 5.66)	0.41
Cough	74 (25.1%)	11 (23.4%)	0.93 (0.54; 1.62)	0.81
Vomiting	10 (3.4%)	2 (4.3%)	1.26 (0.28; 5.55)	0.76

*CI = Confidence Interval; ^†^P-value = Significance level; ^‡^Missing 21; ^§^Value referring to Fisher’s exact test

**Table 5:** Description of long COVID symptoms, considering the doses administered in the patients’ vaccination schedule. São Carlos, SP, Brazil, 2024

Symptoms of long COVID	Vaccine doses administered against COVID-19					
	0 (n = 47)	1 (n = 47)	2 (n = 96)	3 (n = 86)	4 (n = 53)	5 (n = 13)
Changes in menstruation	2 (4.8%)	1 (2.4%)	4 (4.4%)	0 (0%)	1 (1.9%)	0 (0%)
Anxiety	3 (7.1%)	6 (14.3%)	6 (6.6%)	5 (6.0%)	1 (1.9%)	0 (0%)
Fatigue	4 (8.5%)	9 (19.1%)	16 (16.7%)	18 (20.9%)	9 (17.0%)	1 (7.7%)
Runny nose	10 (21.3%)	5 (10.9%)	9 (9.4%)	9 (10.5%)	13 (24.5%)	0 (0%)
Diarrhea	0 (0%)	0 (0%)	2 (2.1%)	2 (2.3%)	0 (0%)	0 (0%)
Difficulty concentrating	1 (2.4%)	0 (0%)	3 (3.3%)	0 (0%)	1 (1.9%)	0 (0%)
Abdominal pain	2 (4.8%)	3 (7.1%)	3 (3.3%)	9 (10.8%)	2 (3.8%)	2 (16.7%)
Joint pain*	1 (2.4%)	1 (2.4%)	3 (3.3%)	2 (2.4)	0 (0%)	0 (0%)
Headache	13 (27.7%)	14 (29.8%)	30 (31.3%)	23 (26.7%)	13 (24.5%)	3 (23.1%)
Sore throat	12 (25.5%)	9 (19.1%)	16 (16.7%)	14 (16.3%)	10 (18.9%)	4 (30.8%)
Pain in lower limbs*	2 (4.8%)	6 (14.3%)	10 (11.0%)	10 (12.0%)	9 (17.3%)	1 (8.3%)
Pain in upper limbs*	1 (2.4%)	3 (7.1%)	6 (6.6%)	2 (2.4%)	3 (5.8%)	0 (0%)
Muscle pain	6 (12.8%)	7 (14.9%)	17 (17.7%)	11 (12.8%)	9 (17.0%)	1 (7.7%)
Back/lower back pain*	3 (7.1%)	5 (11.9%)	6 (6.6%)	7 (8.4%)	8 (15.4%)	0 (0%)
Ear pain	0 (0%)	0 (0%)	1 (1.1%)	0 (0%)	0 (0%)	0 (0%)
Chest pain	6 (14.3%)	2 (4.8%)	3 (3.3%)	2 (3.8%)	2 (3.8%)	1 (8.3%)
Thoracic pain	3 (7.1%)	2 (4.8%)	4 (4.4%)	2 (2.4%)	2 (3.8%)	0 (0%)
Shortness of breath	7 (14.9%)	3 (6.4%)	17 (17.7%)	17 (19.8%)	11 (20.8%)	0 (0%)
Tingling/throbbing in lower limbs	4 (9.5%)	3 (7.1%)	5 (5.5%)	2 (2.4%)	0 (0%)	1 (8.3%)
Tingling/throbbing in upper limbs	1 (2.4%)	2 (4.8%)	4 (4.4%)	2 (2.4%)	1 (1.9%)	1 (8.3%)
Weakness/loss of muscle strength	3 (7.1%)	8 (19.0%)	8 (8.8)	4 (4.8%)	7 (13.5%)	0 (0%)
Insomnia	3 (7.1%)	3 (7.1%)	5 (5.5%)	4 (4.8%)	2 (3.8%)	1 (8.3%)
Fever	4 (8.5%)	5 (10.6%)	8 (8.3%)	10 (11.6%)	5 (9.4%)	2 (15.4%)
Nausea	1 (2.1%)	0 (0%)	3 (3.1%)	1 (1.2%)	3 (5.7%)	0 (0%)
Odynophagia*	1 (2.4%)	0 (0%)	0 (0%)	0 (0%)	0 (0%)	1 (8.3%)
Memory loss/confusion*	1 (2.4%)	0 (0%)	4 (4.4%)	6 (7.2%)	1 (1.9%)	2 (16.7%)
Loss of smell	2 (4.3%)	1 (2.1%)	1 (1.0%)	2 (2.3%)	0 (0%)	1 (7.7%)
Loss of taste	1 (2.1%)	1 (2.1%)	0 (0%)	1 (1.2%)	0 (0%)	1 (7.7%)
Hair loss*	4 (9.5%)	4 (9.5%)	8 (8.8%)	7 (8.4%)	6 (11.5%)	1 (8.3%)
Dental complaint of tooth loss after COVID*	0 (0%)	0 (0%)	1 (1.1%)	2 (2.4%)	0 (0%)	0 (0%)
Sadness/depression*	2 (4.8%)	3 (7.1%)	3 (3.3%)	3 (3.6%)	3 (5.8%)	0 (0%)
Dizziness*	3 (7.1%)	2 (4.8%)	5 (5.5%)	4 (4.8%)	1 (1.9%)	0 (0%)
Cough	11 (23.4%)	12 (25.5%)	26 (27.1%)	20 (23.3%)	12 (22.6%)	4 (30.8%)
Vomiting	2 (4.3%)	0 (0%)	5 (5.2%)	2 (2.3%)	2 (3.8%)	1 (7.7%)
Others^†^	8 (19.0%)	10 (23.8%)	22 (24.4%)	18 (22.0%)	16 (31.4%)	4 (33.3%)

*Missing 20; ^†^Missing 23

## Discussion

This study revealed a higher prevalence of shortness of breath among individuals aged 60 or older, a higher prevalence of hair loss as a symptom of long COVID among women, and a higher prevalence of chest pain among unvaccinated individuals.

Although the end of the global health emergency caused by COVID-19 was declared by the World Health Organization (WHO) in May 2023, the virus continues to spread worldwide. In addition to the risk of new outbreaks, people who have been infected with SARS-CoV-2 pose a challenge to global public health due to symptomatic manifestations that persist or arise after the acute phase of infection^13^.

The pathophysiological mechanisms of long COVID are still being clarified, and evidence indicates that the condition may result from multiple factors. Among the leading hypotheses for its pathogenesis are immune dysregulation, with the possible persistence of viral reservoirs in tissues, metabolic dysregulation, the impact of infection on the microbiota, endothelial dysfunction, autoimmune processes, and post-intensive care syndrome^14^ ^-^ ^15^.

It has been observed that SARS-CoV-2 ribonucleic acid (RNA) can persist for weeks in patients who have clinically recovered from COVID-19, being detected in the respiratory tract, gastrointestinal tract, blood, and cerebrospinal fluid^13^. This long-term inflammatory persistence may be justified by the presence of residual virus antigens, which lead to the persistent activation of T cells. This process can last up to 6 months after the acute infection^16^.

Concomitantly, according to the immunological profile of patients who recovered from SARS-CoV-2, it was possible to evidence the continuous presence of inflammation, vascular damage, and immune cell differentiation in the period from two to eight months after infection. These patients had increased levels of cytokines compared to healthy individuals, especially interleukin 6 (IL-6), which can cross the blood-brain barrier^14^.

In addition, long COVID may be associated with a state of generalized hypersensitivity, which would explain the multiplicity of symptoms reported by patients. Among the most frequent symptoms are pain and cough, which have similar mechanisms of control and peripheral sensitization in their respective afferent pathways. Similarly, chronic fatigue syndrome has also been linked to changes in pain processing and neurogenic sensory sensitization, both peripheral and central. In this context, it is observed that the insular and cingulate cortex, areas involved in the nociceptive processing of dyspnea, are also activated by pain and coughing^17^.

It should be noted that the findings of this study corroborate the literature regarding the symptoms most frequently observed in long COVID, with cough and pain-especially sore throat and headache-standing out as the most prevalent symptoms, regardless of vaccination status, being reported by both unvaccinated individuals and those who have been fully vaccinated against COVID-19.

Regarding the pathophysiology of headaches in long COVID, one hypothesis suggests that SARS-CoV-2 invasion of the central nervous system directly affects neural pathways. Studies have shown a reduction in substances in the parahippocampal gyrus and orbitofrontal cortex in patients with COVID-19, in addition to the presence of elevated levels of cytokines and interleukins in the blood, which may promote persistent immune activation. However, the prolonged headache observed in cases of long COVID still represents a gap in scientific knowledge, requiring further investigation for complete clarification^18^.

As for neuro-otorhinolaryngological symptoms in long COVID, such as sore throat and cough, the explanation may be related to coagulation problems, immune-mediated injury, hypoxia resulting from respiratory failure, and direct viral invasion of tissues. SARS-CoV-2 can cause direct damage by binding to the angiotensin-converting enzyme 2 receptor, commonly expressed in the upper airways, including the nasal, laryngeal, and tracheal tracts, as well as in the neuro-olfactory epithelium and lungs^19^.

According to the study’s results, women were more susceptible to developing long COVID. According to the National Center for Health Statistics, long COVID affects twice as many women as men, with an elevated risk in premenopausal women. This evidence suggests that sex hormones have a potential role in the development of long COVID^20^.

Thus, the entry of SARS-CoV-2 can be facilitated by androgens through the enzyme Transmembrane Serine Protease 2 (TMPRSS2). Additionally, androgen receptors are located on the X chromosome, which determines the biological sex of females. Thus, circulating androgens promote the transcription of TMPRSS2, which prepares SARS-CoV-2 to interact with Angiotensin-Converting Enzyme 2 (ACE 2) receptors, allowing its entry into host cells^6^.

Through androgen coding, a single copy of the X chromosome is produced, allowing for the correlation of different levels of androgen sensitivity. Thus, through these modifications, there is an increased risk of androgen-mediated diseases, such as alopecia. Concurrently with the analysis of this study, it was possible to identify that women had a higher prevalence of hair loss. In summary, the actions mediated by the androgen enzyme TMPRSS2 may explain the differences between the sexes in relation to the outcomes of SARS-CoV-2 infection^6^.

In this study, when addressing age as a variable associated with the occurrence of long COVID, it was evident that older people had a higher prevalence of shortness of breath. In this context, the literature has pointed out that the clinical findings and characteristics of long COVID may differ between age groups^21^ ^-^ ^22^. One analysis showed that participants over 65 years of age had a higher frequency of long COVID symptoms^21^. Although long COVID symptoms such as fatigue and dyspnea are common in both the adult and elderly populations, other symptoms, such as headache, chest pain, palpitations, impaired concentration, and emotional distress, were more prevalent among younger people. In contrast, cough and arthralgia were more common in older people^21^ ^-^ ^22^.

In this scenario, it is observed that the symptoms manifested in long COVID may vary according to age group. One hypothesis for this variation is related to the damage suffered by the body, considering the severity of acute COVID-19 infection. Another possible explanation involves physiological differences between age groups. In this sense, conditions such as reduced reserve and sarcopenia, associated with the aging process, may have different effects and consequences after a viral infection - such as the more frequent presence of symptoms with fatigue and dyspnea in older adults^21^.

It should be noted that many symptoms of long COVID in older adults can be challenging to diagnose, as they may overlap with other health conditions or be associated with the aging process^21^ ^-^ ^23^. Additionally, the diversity of symptoms is another factor that complicates the diagnosis of long COVID. Thus, given the uncertainty regarding the attribution of symptoms to long COVID, in addition to the patient’s report in the clinical evaluation, it may be necessary to perform complementary tests and refer the patient to specialists to assist in defining the diagnosis and directing appropriate treatment^24^.

In this context, it is essential to highlight that all people who have had a COVID-19 infection, regardless of severity, are susceptible to long COVID, with vaccination against COVID-19 being a factor strongly associated with reducing the likelihood of developing persistent symptoms. In this study, it was found that unvaccinated people had a higher prevalence of chest pain as a symptom of long COVID. 

The symptom of chest pain after COVID-19 infection is commonly related to the severity of the disease in its acute phase, resulting from conditions such as prolonged use of invasive mechanical ventilation, acute respiratory distress syndrome, myocarditis, pericarditis, multilobar pneumonia, and pulmonary embolism^25^.

In this context, it is well established that vaccination against COVID-19 is crucial for preventing severe forms of the viral infection during the acute phase. The literature indicates that people who have been infected with the disease and who were not vaccinated are at greater risk of cardiovascular events after viral infection^26^. Thus, the chest symptoms of long COVID could be explained by the location of the systems or organs that suffered damage during the acute phase of the disease^25^ ^-^ ^26^.

In the general context of long COVID symptoms, an analysis in the United Kingdom, Estonia, and Spain showed that one dose of the COVID-19 vaccine was associated with a reduced risk of developing long COVID^27^. On the other hand, another analysis indicated that people who received one dose of the vaccine did not acquire protection against long COVID, while those who received two doses did. Furthermore, people vaccinated against COVID-19 had a lower risk of cognitive dysfunction, kidney problems, myalgia, and sleep disorders^28^.

In another study in Brazil, it was found that incomplete vaccination against COVID-19 was associated with a higher probability of developing long COVID. In addition, the frequency of symptoms such as fatigue and headaches was higher among people with incomplete vaccination schedules^29^. It is worth noting that vaccination reduces the risk of developing long COVID, both in individuals who were vaccinated before contracting COVID-19 and in those who were immunized after contracting the disease^28^.

In this context, vaccination against COVID-19 directly increases antibody titers. It eliminates possible viral reservoirs in the body, thereby reducing the severity of acute SARS-CoV-2 infection-a known risk factor for the development of long COVID^30^ ^-^ ^33^.

The diversity of symptoms that can be attributed to long COVID, combined with the difficulty in defining the diagnosis, poses a challenge for healthcare services. In this sense, adherence to COVID-19 vaccination is the most effective way to prevent long COVID^23^ ^,^ ^28^ ^-^ ^29^ ^,^ ^34^.

Therefore, the clinical evaluation of patients must include the reporting of subjective symptoms, history of previous COVID-19 infection, and the existence of risk factors that may influence the manifestation of long COVID symptoms. Healthcare can thus be tailored to each individual, and a multidisciplinary approach that considers both the physical and psychological aspects of each person is recommended to restore their health and functional status^23^ ^,^ ^34^.

This study makes a significant contribution to health surveillance by identifying population groups that are most vulnerable to long COVID, particularly the elderly, women, and unvaccinated individuals. The findings highlight the importance of continuous monitoring of patients infected with COVID-19 in PHC, in addition to providing support for more targeted and effective health surveillance. In addition, this analysis stands out for its simultaneous approach to gender, age group, and vaccination status as predictors of prolonged COVID-19 symptoms in a population served by the public health system. Furthermore, the use of data from official systems lends methodological robustness to the analysis and broadens the potential applicability of the results to public health management.

Thus, the results obtained can provide essential insights for clinical practice by highlighting the risk factors for long COVID, directly contributing to the improvement of health services - especially PHC and health surveillance - through the formulation of clinical protocols aimed at early detection of symptoms and risk stratification based on sociodemographic and clinical variables. Such measures contribute to more effective care, enhanced surveillance, and improved utilization of available resources.

In the field of nursing, the findings underscore the importance of strategic action by professionals in clinical surveillance, reception, and ongoing monitoring of individuals who present with persistent symptoms following COVID-19 infection. The study’s evidence supports practices such as qualified listening and care planning, enabling a more proactive and individualized approach. Recognizing the most frequent symptoms, valuing subjective reports, and considering factors such as gender, age, and vaccination contribute to more humanized, evidence-based care that is aligned with scientific evidence.

Despite its relevant contributions, this study has some limitations that should be considered when interpreting the results. The primary restriction is the reliance on secondary data from electronic medical records, which may contain incomplete or inconsistent information. This limitation may have led to underreporting of symptoms or omission of relevant clinical information, compromising the accuracy in identifying cases of long COVID. Another limitation of the study concerns the sample size, which does not capture the diversity of the Brazilian population. In addition, the cross-sectional design adopted does not allow for establishing causal relationships between the factors analyzed and the development of long COVID, restricting the analysis to statistical associations.

The use of information from only one municipality also limits the generalization of findings to other regions, especially considering regional inequalities in access to healthcare, PHC structure, and vaccination coverage in Brazil. Additionally, the definition of long COVID used, although based on the WHO proposal, depends on the presence of self-reported symptoms and the patient’s return to the health service after the acute phase, which may exclude individuals who, even with persistent symptoms, did not seek care.

## Conclusion

This study indicated that older people had a higher prevalence of shortness of breath; women had a higher prevalence of hair loss as a symptom of long COVID; and unvaccinated individuals had a higher prevalence of chest pain. Determining the most prevalent symptoms of long COVID, considering age group, gender, and vaccination status, can contribute to the clinical evaluation of the disease, as well as to the identification of possible risk factors for some symptoms manifested in the disease, assisting in the development of protocols that guide the diagnosis and clinical management of the disease.

The findings also highlight the importance of vaccination against COVID-19 as a preventive measure not only against severe forms of acute disease but also against prolonged manifestations. In this sense, strategies to expand vaccination coverage and reinforce public campaigns are essential to reduce the impact of long COVID on the population.

Finally, although the study was conducted in a single municipality, its results apply to similar contexts, such as medium-sized cities with comparable healthcare organizations. Nevertheless, multicenter studies, longitudinal methods, and the incorporation of social and behavioral variables, as well as representative samples from different regions of the country, are recommended to validate the findings and deepen knowledge about the factors associated with long COVID in the Brazilian context.

## Data Availability

Datasets related to this article will be available upon request to the corresponding author.
