# Supplementary material for: Predictors of Long COVID: older adults, women, and an incomplete COVID-19 vaccination schedule
Source: Rev Lat Am Enfermagem. 2026 Jul 20;34:e4816. doi: 10.1590/1518-8345.7954.4816 (PMC13387775; doi:10.1590/1518-8345.7954.4816)
Supplement: Translation

# Translation: Preditores da COVID Longa: pessoas idosas, mulheres e esquema vacinal incompleto contra COVID-19^*^

**DOI:** 10.1590/1518-8345.7954.4817

**Authors:** Karina Marques Prediger, Ana Cristina Ribeiro La Scaléa, Silvia Carla da Silva André Uehara

**Affiliations:** 1 Universidade Federal de São Carlos, Departamento de Enfermagem, São Carlos, SP, Brasil.; 2 Bolsista da Fundação de Amparo à Pesquisa do Estado de São Paulo (FAPESP), Brasil.; 3 Bolsista da Coordenação de Aperfeiçoamento de Pessoal de Nível Superior (CAPES), Brasil.

**Keywords:** Síndrome de COVID-19 Pós-Aguda, Prevalência, Feminino, Fatores Etários, Vacinas, Sinais e Sintomas

## Abstract

**Objetivo:**

analisar os sintomas de COVID Longa em pacientes assistidos na Atenção Primária à Saúde, considerando sexo, idade e esquema vacinal.

**Método:**

trata-se de um estudo transversal e analítico, realizado com uma amostra de 350 pacientes com COVID Longa, atendidos em serviços da Atenção Primária à Saúde. Os dados foram coletados por meio da análise de prontuários e analisados por meio do modelo de regressão de Poisson.

**Resultados:**

pessoas idosas apresentaram 88% maior prevalência de falta de ar como sintoma de COVID Longa; pessoas do sexo feminino apresentaram prevalência 13,5 vezes maior de queda de cabelo; ainda, pessoas não vacinadas apresentaram 3,64 vezes maior prevalência de referir dor no peito.

**Conclusão:**

a identificação dos sintomas da COVID Longa mais prevalentes, considerando a faixa etária, sexo e situação vacinal, pode contribuir na avaliação clínica e na elaboração de protocolos que orientem o manejo adequado dessas pessoas.

## Destaques

**(1)** Pessoas idosas apresentaram maior prevalência de falta de ar na CL. **(2)** Mulheres apresentaram maior prevalência de apresentar queda de cabelo na CL. **(3)** Pessoas não vacinadas apresentaram maior prevalência de referir dor no peito na CL.

## Introdução 

A COVID Longa é caracterizada por um histórico de infecção por COVID-19, cujos sintomas apresentados persistem ou flutuam por, ao menos, dois meses após a fase aguda e não podem ser justificados por um diagnóstico alternativo. Dentre os sintomas mais frequentes de COVID Longa, destacam-se fadiga, falta de ar, disfunção cognitiva, além de outros que impactam nas atividades diárias do paciente^1^. 

Estima-se que a prevalência global da COVID Longa seja, aproximadamente, de 36% e que a prevalência varie conforme a região geográfica, tendo sido estimada em 35% na Ásia, 39% na Europa, 30% na América do Norte e 51% na América do Sul^2^. No Brasil, observa-se também uma variação regional na prevalência da COVID Longa. Entre março de 2020 e março de 2023, os estados com os maiores coeficientes por 100 mil habitantes foram Goiás, Distrito Federal, Paraná, Rio Grande do Sul, Tocantins e São Paulo^3^. 

No período de setembro de 2021 a dezembro de 2024, foram registrados 167.138 atendimentos individuais na Atenção Primária à Saúde (APS) atribuídos como condição de saúde posterior à COVID-19 não especificada (CID-U09.9). O estado de São Paulo apresentou a maior incidência acumulada de atendimento pós-COVID do país, com 2.026,9 atendimentos por 100 mil habitantes, seguido pelos estados do Amazonas, Rio Grande do Sul, Minas Gerais e Tocantins^3^. 

Deste modo, apesar de os coeficientes da COVID Longa apresentarem uma variação em nível global e nacional, é possível observar que uma parcela dos pacientes infectada pela COVID-19 pode desenvolver os sintomas prolongados^4^. Assim, a COVID Longa consiste em uma condição que abrange manifestações heterogêneas e multissistêmicas, envolvendo diferentes sistemas orgânicos, como cardiovascular, gastrointestinal, endócrino e neuropsiquiátrico^5^ ^-^ ^7^. Dessa forma, o profissional de saúde que atende um paciente com esses sintomas pode, facilmente, relacioná-los a causas psicossomáticas, como depressão e ansiedade, o que pode dificultar o diagnóstico da COVID Longa^4^. 

Cabe destacar que pacientes que desenvolveram a forma grave da COVID-19 tendem a manifestar sintomas prolongados após a fase aguda da doença. Nesse contexto, foi verificada a prevalência de sintomas persistentes em pacientes tratados ambulatorialmente, sendo que de 5% a 26% dessas pessoas apresentaram sintomas prolongados de COVID-19 por um período de um a três meses, e de 2% a 62% apresentaram sintomas por um período de três a seis meses após o diagnóstico da doença aguda^8^. 

Já a fisiopatologia da COVID Longa pode estar relacionada a diferentes mecanismos, de acordo com o sistema orgânico afetado. Assim, o desenvolvimento da COVID Longa envolve sequelas contínuas de tecidos gravemente danificados, persistência do SARS-CoV-2 nos órgãos, desregulação do sistema imunológico que desencadeia autoimunidade e inflamação crônica, hipóxia tecidual e dano endotelial^5^. 

Embora a literatura apresente a descrição dos sintomas manifestados na COVID Longa^1^ ^,^ ^4^, dada a semelhança com outras doenças, caracterizar o quadro sintomatológico mais prevalente, de acordo com o sexo, faixa etária e situação vacinal contra a COVID-19, pode auxiliar na identificação do grupo populacional mais propenso a desenvolver tal condição. Dessa forma, esse estudo tem como objetivo analisar os sintomas de COVID Longa em pacientes assistidos na Atenção Primária à Saúde, considerando sexo, idade e esquema vacinal. 

## Método 

### Tipo do estudo 

Trata-se de um estudo transversal e analítico, elaborado de acordo com as recomendações do *Strengthening The Reporting of Observational Studies in Epidemiology* (STROBE)^9^. 

### Cenário 

Este estudo foi realizado no município de São Carlos, localizado no estado de São Paulo. Os dados foram obtidos junto aos serviços de Vigilância em Saúde e ao Departamento de Gestão e Cuidado Ambulatorial, setor responsável pelos serviços da Atenção Primária à Saúde (APS). 

### Período 

Para o cálculo amostral, foram utilizados os dados referentes aos casos de COVID-19 na população adulta notificados no período de 26/02/2020 a 26/02/2024. Assim, foi possível identificar, nesse intervalo, o diagnóstico CID-Z20 (COVID-19 confirmada) e, ao menos, um retorno em serviço da APS após dois meses da data da infecção aguda por COVID-19. A coleta de dados ocorreu entre os meses de agosto de 2023 a junho de 2024. 

### População e amostra do estudo 

Foi realizado um cálculo amostral considerando todos os casos de COVID-19 diagnosticados na população com idade superior a 18 anos no município, referente ao período de 26/02/2020 a 26/02/2024, por meio de Teste Rápido (TR) ou *Reverse Transcription Polymerase Chain Reaction* (RT-PCR). Na cidade de São Carlos-SP, foram notificados 62.977 casos positivos de COVID-19 em indivíduos acima de 18 anos durante o período de análise deste estudo. A escolha da prevalência baseou-se em dados da literatura^10^, que indicam que cerca de 50% dos casos de COVID-19 apresentaram sintomas de COVID Longa. 

Para o cálculo amostral, assumiu-se um nível de significância de 5% (α = 0,05), erro absoluto tolerável de 5 pontos percentuais (*d* = 0,05) e uma população finita estimada em *N* = 31.489 pacientes com COVID Longa acima de 18 anos no município de interesse. A prevalência esperada utilizada foi p = 0,356, correspondente à frequência do sintoma fadiga, uma das manifestações mais relatadas em estudos prévios sobre COVID Longa. Assim, o tamanho amostral adotado neste estudo foi de n = 350. O cálculo foi realizado por meio da seguinte equação:

$$n=\frac{z_{\left(1-\frac{\alpha }{2}\right)}^{2}Np\left(1-p\right)}{d^{2}\left(N-1\right)+z_{\left(1-\frac{\alpha }{2}\right)}^{2}p\left(1-p\right)}$$

Onde *p* é a prevalência esperada, *d* é o erro absoluto tolerável, *N* é o tamanho da população e *z* é o valor da distribuição normal padrão correspondente ao nível de confiança desejado. 

### Critérios de seleção

Foram incluídos no estudo pacientes adultos acima de 18 anos que apresentaram teste positivo para COVID-19 e retornaram aos serviços da APS com sintomas de COVID Longa, conforme a definição da OMS, que descreve os sintomas como sendo reincidentes ou flutuantes por um período mínimo de dois meses após a infecção pela doença aguda^1^. Foram excluídos do estudo pacientes cujo prontuário não estava acessível, que não residiam em localidades adscritas à APS ou que faleceram. 

### Variáveis do estudo 

As variáveis do estudo foram sexo, idade, raça/cor, comorbidades, esquema vacinal e os sintomas relatados. Dentre os sintomas relatados, incluem-se alteração na menstruação, ansiedade, cansaço, coriza, diarreia, dificuldade de concentração, dores: abdominal, articular, de cabeça, garganta, membros inferiores (MMII), membros superiores (MMSS), muscular, nas costas/lombar, ouvido, peito, tórax, falta de ar, febre, formigamento em MMII e/ou MMSS. Além disso, há fraqueza/perda de força muscular, insônia, náusea, odinofagia, perda de memória/confusão, perda de olfato, perda de paladar, queda de cabelo, queixa odontológica de perda dentária, tontura, tosse, tristeza/depressão e vômito. Este estudo considerou sintomas autorrelatados compatíveis com a definição da OMS^1^ para COVID longa, ou seja, com início geralmente três meses após a fase aguda da infecção, duração mínima de dois meses e sem outra explicação clínica identificável.

### Instrumentos utilizados para a coleta de dados das informações 

Para a coleta de dados, foram construídas planilhas no Microsoft Excel, contendo as variáveis de interesse. Inicialmente, foram criadas diferentes abas para a coleta dos dados provenientes dos diferentes bancos utilizados nesta pesquisa, obtidos no SisCOVID, sistema de notificação de COVID-19 do município de São Carlos-SP. Na aba seguinte, foram inseridos os dados referentes à situação vacinal, obtidos no VaciVida, sistema desenvolvido pelo Estado de São Paulo. Por fim, na última aba, foram inseridos os dados referentes aos sintomas de COVID Longa obtidos no Prontuário Eletrônico do Cidadão (PEC). Foram adotadas medidas para minimizar vieses, incluindo o treinamento dos coletores de dados - dois graduandos, dois mestrandos e um doutorando em Ciência da Saúde -, a utilização de critérios de inclusão e exclusão previamente definidos e a padronização da coleta de dados por meio de uma planilha estruturada e digitalizada. Além disso, destacam-se a escolha do nível de significância adequado (α = 0,05), ajuste para múltiplas comparações, a aplicação de métodos estatísticos apropriados e tamanho amostral robusto. 

### Coleta de dados 

Primeiramente, foi realizada a identificação dos casos confirmados de COVID-19 no SisCOVID; posteriormente, consultou-se no PEC se o paciente infectado pelo SARS-CoV-2 apresentou retorno na APS com relato de sintomas de COVID Longa, de acordo com a definição da OMS^1^. Por meio do SisCOVID, também foi possível obter informações sobre consultas de usuários com queixas de sintomas respiratórios após a fase aguda da doença; já pelo PEC, verificaram-se os sintomas de COVID Longa referidos pelos usuários, que atingiam os diferentes sistemas orgânicos do corpo. Além disso, os dados referentes à vacinação foram obtidos na página do VaciVida, junto à vigilância epidemiológica municipal.

### Tratamento e análise dos dados 

Inicialmente, os dados foram descritos por meio de frequências absolutas e percentuais (variáveis qualitativas) e por medidas de centralidade e dispersão, como média, desvio-padrão, mínimo, mediana e máximo (variáveis quantitativas). Para estimar a razão de prevalência bruta dos sintomas de COVID Longa em relação às variáveis de interesse, foi utilizado o modelo de regressão de Poisson com variância robusta^11^. Nas análises em que a frequência dos sintomas era nula, foi realizado o teste exato de Fisher. O uso de *Missing Data* foi indicado no rodapé das tabelas para garantir a transparência dos dados apresentados, mantendo-se o número de casos ausentes conforme observados, sem imputação. Todas as análises foram realizadas com o auxílio do *software* SAS 9.4^12^, adotando-se um nível de significância de 5%. 

### Aspectos éticos 

Conforme a Resolução n.º 510/2016, o projeto foi aprovado pelo Comitê de Ética em Pesquisa da Universidade Federal de São Carlos (CAAE: 67128023.4.0000.5504; CAAE: 74911623.5.0000.5504). 

## Resultados 

Foram analisados 3.778 casos notificados e confirmados de COVID-19 para alcançar a amostra de 350 casos de COVID Longa, o que corresponde a 12% dos casos da doença aguda na população acima de 18 anos do município de São Carlos-SP. A amostra do estudo é composta por 67,1% (235) do sexo feminino e 32,9% (115) do sexo masculino; 61,6% (215) se declararam de cor branca, 30,1% (105) de cor parda, 8,0% (28) de cor preta, 0,3% (1) de cor amarela, e 1 caso não teve a raça/cor registrada. Quanto à faixa etária, 77,1% (270) eram pessoas com idade inferior a 60 anos e 22,9% (80), pessoas com idade igual ou superior a 60 anos. A idade média foi de 46,9 anos (Tabela 1). 

Ao comparar os sintomas de COVID Longa apresentados pelos pacientes, considerando o sexo, destaca-se que pessoas do sexo feminino apresentaram 13,5 vezes maior prevalência de queda de cabelo em comparação às do sexo masculino (Tabela 2). 

**Tabela 1:** Caracterização demográfica e clínica da amostra do estudo. São Carlos, SP, Brasil, 2024

Características	Frequência (n = 350)	Percentual (%)
**Sexo**		
Feminino	235	67,1
Masculino	115	32,9
**Idade**		
Média	46,9 [15,8]	-
Mediana (Mín.* e Máx.^†^)	47,0 [14,0 e 88,0]	-
**Faixa etária**		
< 60 anos	270	77,1%
≥ 60 anos	80	22,9%
**Raça/Cor**		
Amarela	1	0,3
Branco	215	61,6
Pardo	105	30,1
Preto	28	8,0
*Missing*	1	-
**Comorbidades**		
Hemoglobinopatias	1	0,3
Cirrose hepática	0	0
Doenças cardiovasculares	17	4,9
Doenças neurológicas	4	1,1
Diabetes Mellitus (Tipo 1 e Tipo 2)	53	15,1
Doença Renal Crônica	4	1,1
Hipertensão Arterial Sistêmica	106	30,3
Imunossuprimidos	0	0
Obesidade	56	16
Pneumopatias crônicas graves	21	6,0
Outros	51	14,6

*Mín. = Mínimo; ^†^Máx. = Máximo

**Tabela 2:** Comparação da presença de sintomas de COVID Longa, considerando o sexo da população de estudo. São Carlos, SP, Brasil, 2024

Sintomas de COVID Longa	F* (n = 235)	M^†^ (n = 115)	Razão de prevalência (F* *vs* M ^†^) **[IC 95%]** ^‡^	Valor p^§^
Alteração na menstruação^||^	8 (3,6%)	0 (0%)	-	0,06^¶^
Ansiedade^||^	14 (6,3%)	7 (6,7%)	0,94 (0,39; 2,25)	0,89
Cansaço	44 (18,7%)	13 (11,3%)	1,66 (0,93; 2,95)	0,09
Coriza	32 (13,7%)	14 (12,2%)	1,12 (0,62; 2,02)	0,70
Diarreia	4 (1,7%)	0 (0%)	-	0,31^¶^
Dificuldade de concentração^||^	5 (2,2%)	0 (0%)	-	0,18^¶^
Dor abdominal^||^	13 (58%)	8 (7,6%)	0,76 (0,33; 1,78)	0,53
Dor articular^||^	4 (1,8%)	3 (2,9%)	0,63 (0,14; 2,74)	0,53
Dor de cabeça	72 (30,6%)	25 (21,7%)	1,41 (0,95; 2,1)	0,09
Dor de garganta	46 (19,6%)	20 (17,4%)	1,13 (0,7; 1,81)	0,63
Dor em membros inferiores^||^	24 (10,7%)	15 (14,3%)	0,75 (0,41; 1,37)	0,35
Dor em membros superiores^||^	9 (4,0%)	6 (5,7%)	0,7 (0,26; 1,92)	0,49
Dor muscular	37 (15,7%)	14 (12,2%)	1,29 (0,73; 2,29)	0,38
Dor nas costas/lombar^||^	22 (9,8%)	8 (7,6%)	1,29 (0,59; 2,8)	0,52
Dor no ouvido^||^	1 (0,4%)	0 (0%)	-	0,99^¶^
Dor no peito^||^	10 (4,5%)	7 (6,7)	0,67 (0,26; 1,71)	0,40
Dor no tórax^||^	10 (4,5%)	3 (2,9%)	1,56 (0,44; 5,56)	0,49
Falta de ar	33 (14,0%)	23 (20,0%)	0,7 (0,43; 1,14)	0,15
Febre	26 (11,1%)	8 (7,0%)	1,59 (0,74; 3,4)	0,23
Formigamento em membros inferiores^||^	11 (4,9%)	5 (4,8%)	1,03 (0,37; 2,89)	0,95
Formigamento em membros superiores^||^	10 (4,5%)	1 (1,0%)	4,69 (0,61; 36,14)	0,14
Fraqueza/perda da força muscular^||^	21 (9,4%)	9 (8,6%)	1,09 (0,52; 2,31)	0,81
Insônia^||^	15 (6,7%)	4 (3,8%)	1,76 (0,6; 5,17)	0,31
Náusea	7 (3,0%)	1 (0,9%)	3,43 (0,43; 27,51)	0,25
Odinofagia^||^	1 (0,4%)	1 (1,0%)	0,47 (0,03; 7,42)	0,59
Perda de memória/confusão^||^	10 (4,5%)	5 (4,8%)	0,94 (0,33; 2,67)	0,90
Perda de olfato	3 (1,3%)	4 (3,5%)	0,37 (0,08; 1,61)	0,18
Perda de paladar	3 (1,3%)	1 (0,9%)	1,47 (0,15; 13,96)	0,74
Queda de cabelo^||^	29 (12,9%)	1 (1,0%)	13,59 (1,88; 98,45)	0,01
Queixa odontológica de perda dentária^||^	2 (0,9%)	1 (1,0%)	0,94 (0,09; 10,22)	0,96
Tontura	11 (4,9%)	4 (3,8%)	1,29 (0,42; 3,95)	0,66
Tosse	59 (25,1%)	27 (23,5%)	1,07 (0,72; 1,59)	0,74
Tristeza/depressão^||^	11 (4,9%)	4 (3,8%)	1,29 (0,42; 3,95)	0,66
Vômito	9 (3,8%)	3 (2,6%)	1,47 (0,41; 5,32)	0,56

*F = Sexo feminino; ^†^M = Sexo masculino; ^‡^IC = Intervalo de Confiança; ^§^Valor p = Nível de significância; ^||^ *Missing* 20; ^¶^Valor referente ao teste exato de Fisher

Já ao comparar os sintomas de COVID Longa dos pacientes, considerando a faixa etária, destaca-se que pessoas com idade maior ou igual a 60 anos apresentaram 88% maior prevalência de falta de ar, além de uma diminuição na prevalência de 52% de dor de cabeça, 66% de dor de garganta e 63% de dor muscular em relação às pessoas adultas mais jovens (Tabela 3). 

Os resultados evidenciaram que pessoas com COVID Longa que não receberam nenhuma dose de vacina contra COVID-19 apresentaram 3,64 vezes maior prevalência de dor no peito (Tabela 4).

Os resultados mostram que 27,2% (96) das pessoas com COVID Longa tinham recebido 2 doses da vacina contra a COVID-19, sendo a dor de cabeça o principal sintoma nesse grupo, presente em 31,3% (30) dos casos analisados. Verifica-se que, independentemente da quantidade de doses administradas contra a COVID-19, os sintomas comuns da COVID Longa entre as pessoas foram dor de cabeça, dor de garganta e tosse (Tabela 5).

**Tabela 3:** Comparação da presença de sintomas de COVID Longa, considerando a faixa etária da população de estudo. São Carlos, SP, Brasil, 2024.

Descrição dos sintomas de COVID Longa	< 60 anos (n = 270)	≥ 60 anos (n = 80)	Razão de prevalência (≥ 60 *vs* < 60) [IC 95%]*	Valor p^†^
Alteração na menstruação^‡^	8 (3,2%)	0 (0%)	-	0,21^§^
Ansiedade^‡^	19 (7,6%)	2 (2,6%)	0,34 (0,08; 1,42)	0,14
Cansaço	47 (17,4%)	10 (12,5%)	0,72 (0,38; 1,36)	0,92
Coriza	41 (15,2%)	5 (6,3%)	0,41 (0,17; 1)	0,05
Diarreia	3 (1,1%)	1 (1,3%)	1,13 (0,12; 10,67)	0,92
Dificuldade de concentração^‡^	5 (2,0%)	0 (0%)	-	0,60^§^
Dor abdominal^‡^	16 (6,4%)	5 (6,4%)	1,01 (0,38; 2,66)	0,99
Dor articular^‡^	5 (2,0%)	2 (2,6%)	1,29 (0,25; 6,5)	0,76
Dor de cabeça	85 (31,5%)	12 (15,0%)	0,48 (0,27; 0,83)	<0,01
Dor de garganta	60 (22,2%)	6 (7,5%)	0,34 (0,15; 0,89)	0,03
Dor em membros inferiores^‡^	29 (11,6%)	10 (12,8%)	1,11 (0,57; 2,17)	0,76
Dor em membros superiores^‡^	11 (4,4%)	4 (5,1%)	1,17 (0,38; 3,57)	0,78
Dor muscular	46 (17,0%)	5 (6,3%)	0,37 (0,15; 0,89)	0,03
Dor nas costas/lombar^‡^	23 (9,2%)	7 (9,0%)	0,98 (0,44; 2,19)	0,96
Dor no ouvido^‡^	0 (0%)	1 (1,3%)	-	0,24^§^
Dor no peito^‡^	15 (6,0%)	2 (2,6%)	0,43 (0,1; 1,84)	0,25
Dor no tórax^‡^	12 (4,8%)	1 (1,3%)	0,27 (0,04; 2,03)	0,20
Falta de ar	36 (13,3%)	20 (25,0%)	1,88 (1,15; 3,05)	0,01
Formigamento/latejamento em membros inferiores^‡^	14 (5,6%)	2 (2,6%)	0,46 (0,11; 1,98)	0,30
Formigamento/latejamento em membros superiores^‡^	8 (3,2%)	3 (3,8%)	1,21 (0,33; 4,44)	0,78
Fraqueza/perda da força muscular^‡^	20 (8,0%)	10 (12,8%)	1,61 (0,79; 3,29)	0,19
Insônia^‡^	15 (6,0%)	4 (5,1%)	0,86 (0,29; 2,51)	0,78
Febre	28 (10,4%)	6 (7,5%)	0,72 (0,31; 1,68)	0,45
Náusea	8 (3,0%)	0 (0%)	-	0,21
Odinofagia^‡^	2 (0,8%)	0 (0%)	-	0,99^§^
Perda de memória/confusão^‡^	10 (4,0%)	5 (6,4%)	1,61 (0,57; 4,57)	0,37
Perda de olfato	5 (1,9%)	2 (2,55)	1,35 (0,27; 6,83)	0,72
Perda de paladar	3 (1,1%)	1 (1,3%)	1,13 (0,12; 10,67)	0,92
Queda de cabelo^‡^	27 (10,8%)	3 (3,8%)	0,36 (011; 1,15)	0,08
Queixa odontológica de perda dentária pós-COVID^‡^	2 (0,8%)	1 (1,3%)	1,61 (0,15; 17,51)	0,70
Tristeza/depressão^‡^	11 (4,4%)	4 (5,1%)	1,17 (0,38; 3,57)	0,78
Tontura^‡^	11 (4,4%)	4 (5,1%)	1,17 (0,38; 3,57)	0,78
Tosse	73 (27,0%)	13 (16,3%)	0,6 (0,35; 1,03)	0,06
Vômito	10 (3,7%)	2 (2,5%)	0,68 (0,15; 3,02)	0,61

*IC = Intervalo de Confiança; ^†^Valor p = Nível de significação; ^‡^ *Missing* 21; ^§^Valor referente ao teste exato de Fisher

**Tabela 4:** Comparação da presença de sintomas de COVID Longa, considerando o esquema vacinal da população de estudo. São Carlos, SP, Brasil, 2024

Descrição dos sintomas de COVID Longa	Antes da vacina (n = 295)	Depois da vacina (n = 47)	Razão de prevalência [IC 95%]*	Valor p^†^
Alteração na menstruação^‡^	6 (2,1%)	2 (4,8%)	2,22 (0,46; 10,65)	0,32
Ansiedade^‡^	18 (6,4%)	3 (7,1%)	1,11 (0,34; 3,61)	0,86
Cansaço	53 (18,0%)	4 (8,5%)	0,47 (0,18; 1,25)	0,13
Coriza	36 (12,2%)	10 (21,3%)	1,74 (0,93; 3,26)	0,09
Diarreia	4 (1,4%)	0 (0%)	-	0,99^§^
Dificuldade de concentração^‡^	4 (1,4%)	1 (2,4%)	1,67 (0,19; 14,56)	0,64
Dor abdominal^‡^	19 (6,8%)	2 (4,8%)	0,7 (0,17; 2,9)	0,63
Dor articular^‡^	6 (2,1%)	1 (2,4%)	1,11 (0,14; 9)	0,92
Dor de cabeça	83 (28,1%)	13 (27,7%)	0,98 (0,6; 1,62)	0,95
Dor de garganta	53 (18,0%)	12 (25,5%)	1,42 (0,82; 2,45)	0,21
Dor em membros inferiores^‡^	36 (12,9%)	2 (4,8%)	0,37 (0,09; 1,48)	0,16
Dor em membros superiores^‡^	14 (5,0%)	1 (2,4%)	0,48 (0,06; 3,53)	0,47
Dor muscular	45 (15,3%)	6 (12,8%)	0,84 (0,38; 1,85)	0,66
Dor nas costas/lombar^‡^	26 (9,3%)	3 (7,1%)	0,77 (0,24; 2,43)	0,65
Dor no ouvido^‡^	1 (0,4%)	0 (0%)	-	0,99^§^
Dor no peito	11 (3,9%)	6 (14,3%)	3,64 (1,42; 9,31)	<0,01
Dor no tórax^‡^	10 (3,6%)	3 (7,1%)	2 (0,57; 6,97)	0,28
Falta de ar	48 (16,3%)	7 (14,9%)	0,92 (0,44; 1,9)	0,81
Formigamento/latejamento em membros inferiores^‡^	11 (3,9%)	4 (9,5%)	2,42 (0,81; 7,26)	0,11
Formigamento/latejamento em membros superiores^‡^	10 (3,6%)	1 (2,4%)	0,67 (0,09; 5,08)	0,70
Fraqueza/perda da força muscular^‡^	27 (9,6%)	3 (7,1%)	0,74 (0,24; 2,33)	0,61
Insônia^‡^	15 (5,4%)	3 (7,1%)	1,33 (0,4; 4,41)	0,64
Febre	30 (10,2%)	4 (8,5%)	0,84 (0,31; 2,27)	0,73
Náusea	7 (2,4%)	1 (2,1%)	0,9 (0,11; 7,12)	0,92
Odinofagia^‡^	1 (0,4%)	1 (2,4%)	6,67 (0,43; 104,57)	0,18
Perda de memória/confusão^‡^	13 (4,6%)	1 (2,4%)	0,51 (0,07; 3,82)	0,51
Perda de olfato	5 (1,7%)	2 (4,3%)	2,51 (0,5; 12,57)	0,26
Perda de paladar	3 (1,0%)	1 (2,1%)	2,09 (0,22; 19,69)	0,52
Queda de cabelo^‡^	26 (9,3%)	4 (9,5%)	1,03 (0,38; 2,79)	0,96
Queixa odontológica de perda dentária pós-COVID^‡^	3 (1,11%)	0 (0%)	-	0,99^§^
Tristeza/depressão^‡^	12 (4,3%)	2 (4,8%)	1,11 (0,26; 4,79)	0,89
Tontura^‡^	12 (4,3%)	3 (7,1%)	1,67 (0,49; 5,66)	0,41
Tosse	74 (25,1%)	11 (23,4%)	0,93 (0,54; 1,62)	0,81
Vômito	10 (3,4%)	2 (4,3%)	1,26 (0,28; 5,55)	0,76

*IC = Intervalo de Confiança; ^†^Valor p = Nível de significância; ^‡^ *Missing* 21; ^§^Valor referente ao teste exato de Fisher

**Tabela 5:** Descrição dos sintomas de COVID longa, considerando as doses administradas no esquema vacinal dos pacientes. São Carlos, SP, Brasil, 2024

Sintomas de COVID Longa	Doses de vacina administradas contra COVID-19					
	0 (n = 47)	1 (n = 47)	2 (n = 96)	3 (n = 86)	4 (n = 53)	5 (n = 13)
Alteração na menstruação*	2 (4,8%)	1 (2,4%)	4 (4,4%)	0 (0%)	1 (1,9%)	0 (0%)
Ansiedade*	3 (7,1%)	6 (14,3%)	6 (6,6%)	5 (6,0%)	1 (1,9%)	0 (0%)
Cansaço	4 (8,5%)	9 (19,1%)	16 (16,7%)	18 (20,9%)	9 (17,0%)	1 (7,7%)
Coriza	10 (21,3%)	5 (10,9%)	9 (9,4%)	9 (10,5%)	13 (24,5%)	0 (0%)
Diarreia	0 (0%)	0 (0%)	2 (2,1%)	2 (2,3%)	0 (0%)	0 (0%)
Dificuldade de concentração*	1 (2,4%)	0 (0%)	3 (3,3%)	0 (0%)	1 (1,9%)	0 (0%)
Dor abdominal*	2 (4,8%)	3 (7,1%)	3 (3,3%)	9 (10,8%)	2 (3,8%)	2 (16,7%)
Dor articular*	1 (2,4%)	1 (2,4%)	3 (3,3%)	2 (2,4)	0 (0%)	0 (0%)
Dor de cabeça	13 (27,7%)	14 (29,8%)	30 (31,3%)	23 (26,7%)	13 (24,5%)	3 (23,1%)
Dor de garganta	12 (25,5%)	9 (19,1%)	16 (16,7%)	14 (16,3%)	10 (18,9%)	4 (30,8%)
Dor em membros inferiores*	2 (4,8%)	6 (14,3%)	10 (11,0%)	10 (12,0%)	9 (17,3%)	1 (8,3%)
Dor em membros superiores*	1 (2,4%)	3 (7,1%)	6 (6,6%)	2 (2,4%)	3 (5,8%)	0 (0%)
Dor muscular	6 (12,8%)	7 (14,9%)	17 (17,7%)	11 (12,8%)	9 (17,0%)	1 (7,7%)
Dor nas costas/lombar*	3 (7,1%)	5 (11,9%)	6 (6,6%)	7 (8,4%)	8 (15,4%)	0 (0%)
Dor no ouvido*	0 (0%)	0 (0%)	1 (1,1%)	0 (0%)	0 (0%)	0 (0%)
Dor no peito*	6 (14,3%)	2 (4,8%)	3 (3,3%)	2 (3,8%)	2 (3,8%)	1 (8,3%)
Dor no tórax*	3 (7,1%)	2 (4,8%)	4 (4,4%)	2 (2,4%)	2 (3,8%)	0 (0%)
Falta de ar	7 (14,9%)	3 (6,4%)	17 (17,7%)	17 (19,8%)	11 (20,8%)	0 (0%)
Formigamento/latejamento em membros inferiores*	4 (9,5%)	3 (7,1%)	5 (5,5%)	2 (2,4%)	0 (0%)	1 (8,3%)
Formigamento/latejamento em membros superiores*	1 (2,4%)	2 (4,8%)	4 (4,4%)	2 (2,4%)	1 (1,9%)	1 (8,3%)
Fraqueza/perda da força muscular*	3 (7,1%)	8 (19,0%)	8 (8,8)	4 (4,8%)	7 (13,5%)	0 (0%)
Insônia*	3 (7,1%)	3 (7,1%)	5 (5,5%)	4 (4,8%)	2 (3,8%)	1 (8,3%)
Febre	4 (8,5%)	5 (10,6%)	8 (8,3%)	10 (11,6%)	5 (9,4%)	2 (15,4%)
Náusea	1 (2,1%)	0 (0%)	3 (3,1%)	1 (1,2%)	3 (5,7%)	0 (0%)
Odinofagia*	1 (2,4%)	0 (0%)	0 (0%)	0 (0%)	0 (0%)	1 (8,3%)
Perda de memória/confusão*	1 (2,4%)	0 (0%)	4 (4,4%)	6 (7,2%)	1 (1,9%)	2 (16,7%)
Perda de olfato	2 (4,3%)	1 (2,1%)	1 (1,0%)	2 (2,3%)	0 (0%)	1 (7,7%)
Perda de paladar	1 (2,1%)	1 (2,1%)	0 (0%)	1 (1,2%)	0 (0%)	1 (7,7%)
Queda de cabelo*	4 (9,5%)	4 (9,5%)	8 (8,8%)	7 (8,4%)	6 (11,5%)	1 (8,3%)
Queixa odontológica de perda dentária pós-COVID*	0 (0%)	0 (0%)	1 (1,1%)	2 (2,4%)	0 (0%)	0 (0%)
Tristeza/depressão*	2 (4,8%)	3 (7,1%)	3 (3,3%)	3 (3,6%)	3 (5,8%)	0 (0%)
Tontura*	3 (7,1%)	2 (4,8%)	5 (5,5%)	4 (4,8%)	1 (1,9%)	0 (0%)
Tosse	11 (23,4%)	12 (25,5%)	26 (27,1%)	20 (23,3%)	12 (22,6%)	4 (30,8%)
Vômito	2 (4,3%)	0 (0%)	5 (5,2%)	2 (2,3%)	2 (3,8%)	1 (7,7%)
Outros^†^	8 (19,0%)	10 (23,8%)	22 (24,4%)	18 (22,0%)	16 (31,4%)	4 (33,3%)

*Missing 20; ^†^ *Missing* 23

## Discussão

Este estudo evidenciou maior prevalência de falta de ar entre pessoas com 60 anos ou mais; maior prevalência de queda de cabelo como sintoma de COVID Longa entre pessoas do sexo feminino; e maior prevalência de dor no peito entre indivíduos não vacinados.

Embora o fim da emergência sanitária global causada pela COVID-19 tenha sido declarado pela Organização Mundial de Saúde (OMS) em maio de 2023, o vírus continua disseminado mundialmente. Além do risco de novos surtos, as pessoas que foram infectadas pelo SARS-CoV-2 representam um desafio para a saúde pública global, devido às manifestações sintomáticas que persistem ou surgem após a fase aguda da infecção^13^. 

Os mecanismos fisiopatológicos da COVID Longa ainda estão sendo esclarecidos, e evidências indicam que a condição pode resultar de múltiplos fatores. Entre as principais hipóteses para sua patogênese, destacam-se: a desregulação imunológica com possível persistência de reservatórios virais nos tecidos, desregulação metabólica, impactos da infecção na microbiota, disfunção endotelial, processos autoimunes e a síndrome de tratamento pós-intensivo^14^ ^-^ ^15^.

Observou-se que o Ácido Ribonucleico (RNA) do SARS-CoV-2 pode persistir por semanas em pacientes clinicamente recuperados da COVID-19, sendo detectado no trato respiratório, gastrointestinal, sangue e líquido cefalorraquidiano^13^. Essa persistência inflamatória de longo prazo pode ser justificada pela presença dos antígenos residuais do vírus, ocasionando a ativação persistente das células T, um processo que pode perdurar por até 6 meses após a infecção aguda^16^.

Concomitantemente, de acordo com o perfil imunológico dos pacientes que se recuperaram do SARS-CoV-2, foi possível evidenciar a presença contínua de inflamação, dano vascular e diferenciação de células imunológicas no período de dois a oito meses após a infecção. Esses pacientes apresentaram níveis aumentados de citocinas em relação aos indivíduos saudáveis, especialmente a interleucina 6 (IL-6), capaz de atravessar a barreira hematoencefálica^14^. 

Além disso, a COVID Longa pode estar associada a um estado de hipersensibilidade generalizada, o que explicaria a multiplicidade de sintomas relatados pelos pacientes. Entre os sintomas mais frequentes, destacam-se a dor e a tosse, que apresentam mecanismos semelhantes de controle e sensibilização periférica em suas respectivas vias aferentes. Da mesma forma, a síndrome da fadiga crônica também foi relacionada a alterações no processamento da dor e na sensibilização sensorial neurogênica, tanto periférica quanto central. Nesse contexto, observa-se que o córtex insular e o cingulado, áreas envolvidas no processamento nociceptivo da dispneia, também são ativados pela dor e pela tosse^17^. 

Ressalta-se que os achados deste estudo corroboram a literatura quanto aos sintomas mais frequentemente observados na COVID Longa, destacando-se a tosse e as dores - especialmente dor de garganta e dor de cabeça - como os sintomas mais prevalentes, independentemente do status vacinal, sendo relatados tanto por pessoas não vacinadas quanto por aquelas com esquema vacinal completo contra a COVID-19.

Em relação à fisiopatologia da dor de cabeça na COVID Longa, uma das hipóteses sugere que a invasão do SARS-CoV-2 no sistema nervoso central afeta diretamente as vias neuronais. Estudos apontaram redução de substância no giro parahipocampal e no córtex orbitofrontal em pacientes com COVID-19, além da presença de níveis elevados de citocinas e interleucinas no sangue, os quais podem promover ativação imunológica persistente. No entanto, a cefaleia prolongada observada em casos da COVID Longa ainda representa uma lacuna no conhecimento científico, carecendo de investigações mais aprofundadas para seu completo esclarecimento^18^. 

Já em relação aos sintomas neuro-otorrinolaringológicos na COVID Longa, como dor de garganta e tosse, a explicação pode estar relacionada a problemas de coagulação, lesão imunomediada, hipóxia resultante de insuficiência respiratória e à própria invasão viral direta dos tecidos. O SARS-Cov-2 pode causar danos diretos ao se ligar ao receptor da enzima conversora da angiotensina 2, comumente expresso nas vias aéreas superiores, incluindo os tratos nasal, laríngeo e traqueal, assim como no epitélio neuro-olfatório e nos pulmões^19^. 

De acordo com os resultados deste estudo, as mulheres apresentaram-se mais suscetíveis ao desenvolvimento da COVID Longa. De acordo com o *National Center for Health Statistics*, a COVID Longa afeta duas vezes mais mulheres do que homens, apresentando um risco elevado em mulheres na pré-menopausa. Essas evidências sugerem que os hormônios sexuais apresentam potencialidade no desenvolvimento da COVID Longa^20^. 

Assim, a entrada do SARS-CoV-2 pode ser facilitada pelos andrógenos por meio da enzima Protease Serina Transmembranar 2 (TMPRSS 2). Ademais, os receptores de andrógenos estão localizados no cromossomo X, determinante para o sexo biológico feminino. Assim, o andrógeno circulante promove a transcrição da TMPRSS 2, que prepara o SARS-CoV-2 para interagir com os receptores de Enzima Conversora de Angiotensina 2 (ECA 2), permitindo sua entrada nas células hospedeiras^6^. 

Por meio da codificação dos andrógenos, é realizada uma cópia única do cromossomo X, o que permite correlacionar os diferentes níveis de sensibilidade ao andrógeno. Assim, por meio dessas modificações, há um aumento do risco de doenças que são mediadas pelos andrógenos, como a alopecia. Concomitantemente à análise deste estudo, foi possível identificar que as mulheres apresentaram maior prevalência de queda de cabelo. Em resumo, as ações mediadas pela enzima TMPRSS 2 do andrógeno podem explicar as diferenças entre os sexos em relação aos desfechos da infecção do SARS-CoV-2^6^. 

Neste estudo, ao abordar a idade como uma variável associada à ocorrência da COVID Longa, foi evidenciado que pessoas idosas apresentaram maior prevalência de falta de ar. Nesse contexto, a literatura tem apontado que os achados clínicos e as características da COVID Longa podem diferir entre as faixas etárias^21^ ^-^ ^22^. Uma análise mostrou que os participantes com idade acima de 65 anos apresentaram uma maior frequência de sintomas da COVID Longa^21^. Embora os sintomas da COVID Longa como fadiga e dispneia, sejam comuns tanto na população adulta como na idosa, outros sintomas, como dor de cabeça, dor no peito, palpitações, comprometimento da concentração e sofrimento emocional, foram mais prevalentes entre os mais jovens. Em contrapartida, tosse e artralgia foram mais comuns em pessoas idosas^21^ ^-^ ^22^.

Neste cenário, observa-se que os sintomas manifestados na COVID Longa podem variar de acordo com a faixa etária. Uma hipótese para essa variação está relacionada ao dano sofrido pelo organismo, considerando a gravidade da infecção aguda por COVID-19. Outra possível explicação envolve as diferenças fisiológicas entre as faixas etárias. Nesse sentido, condições como a redução da reserva e sarcopenia, associadas ao processo de envelhecimento, podem apresentar diferentes efeitos e consequências após uma infecção viral - como, por exemplo, a presença mais frequente de sintomas com fadiga e dispneia em pessoas idosas^21^.

Ressalta-se que muitos sintomas da COVID Longa em pessoas idosas podem ter seu diagnóstico dificultado, uma vez que podem se sobrepor a outras condições de saúde ou estarem associados ao processo de envelhecimento^21^ ^-^ ^23^. Além disso, a diversidade de sintomas representa outro fator que dificulta o diagnóstico da COVID Longa. Desta maneira, diante da incerteza quanto à atribuição dos sintomas à COVID Longa, além do relato do paciente na avaliação clínica, pode ser necessária a realização de exames complementares, bem como o encaminhamento a especialistas, para auxiliar na definição do diagnóstico e direcionar o tratamento adequado^24^. 

Neste contexto, é importante destacar que todas as pessoas que tiveram a infecção por COVID-19, independentemente da gravidade, estão suscetíveis à COVID Longa, sendo a vacinação contra a COVID-19 um fator fortemente associado à redução da probabilidade de manifestar os sintomas persistentes. Neste estudo, evidenciou-se que pessoas não vacinadas apresentaram maior prevalência de apresentar dor no peito como sintoma da COVID Longa. 

O sintoma de dor no peito após a infecção por COVID-19 está comumente relacionado à gravidade da doença em sua fase aguda, sendo resultante de condições como uso prolongado de ventilação mecânica invasiva, síndrome do desconforto respiratório agudo, miocardite, pericardite, pneumonia multilobar e embolia pulmonar^25^. 

Nesta conjuntura, é de conhecimento que a vacinação contra a COVID-19 é primordial para a prevenção de formas graves da infecção viral na fase aguda. A literatura aponta que pessoas que foram infectadas pela doença e que não estavam vacinadas apresentam maior risco de ocorrência de eventos cardiovasculares após a infecção viral^26^. Assim, os sintomas torácicos da COVID Longa poderiam ser explicados em decorrência da localização dos sistemas ou órgãos que sofreram danos durante a fase aguda da doença^25^ ^-^ ^26^. 

No contexto geral da manifestação de sintomas da COVID Longa, uma análise no Reino Unido, Estônia e Espanha mostrou que uma dose da vacina contra a COVID-19 foi associada a um risco reduzido de desenvolver COVID Longa^27^. Por outro lado, outra análise evidenciou que pessoas que receberam uma dose da vacina não adquiriram proteção contra a COVID Longa, enquanto aquelas que receberam duas doses a obtiveram. Ainda, pessoas vacinadas contra a COVID-19 apresentaram menor risco de disfunção cognitiva, problemas renais, mialgia e distúrbios do sono^28^.

Em outro estudo no Brasil, foi verificado que esquema de vacinação incompleto contra a COVID-19 foi associado a uma maior probabilidade de desenvolvimento da COVID Longa. Além disso, a frequência de sintomas como fadiga e dores de cabeça foi maior entre pessoas com esquemas de vacinação incompletos^29^. Ressalta-se que a vacinação reduziu o risco de desenvolver a COVID Longa tanto em pessoas que se vacinaram antes da infecção por COVID-19 quanto nas pessoas que se vacinaram após a infecção pela doença^28^.

Nesta conjuntura, a vacinação contra a COVID-19 atua diretamente no aumento dos títulos de anticorpos e na eliminação de possíveis reservatórios virais existentes no organismo, implicando, consequentemente, a redução da gravidade da infecção aguda por SARS-CoV-2 - um fator de risco conhecido para o desenvolvimento da COVID Longa^30^ ^-^ ^33^. 

A diversidade de sintomas que podem ser atribuídos à COVID Longa e a dificuldade na definição do diagnóstico consistem em um desafio para os serviços de saúde. Nesse sentido, a adesão à vacinação contra a COVID-19 consiste na forma mais eficaz para evitar a COVID Longa^23^ ^,^ ^28^ ^-^ ^29^ ^,^ ^34^.

Deste modo, é essencial que a avaliação clínica dos pacientes inclua o relato dos sintomas subjetivos, histórico de infecção prévia por COVID-19 e a existência de fatores de risco que podem influenciar na manifestação dos sintomas da COVID Longa. Assim, os cuidados de saúde podem ser direcionados de forma individualizada, sendo recomendada uma abordagem multidisciplinar que contemple os aspectos físicos e psicológicos de cada indivíduo, com o objetivo de restabelecer a saúde e o estado funcional da pessoa^23^ ^,^ ^34^. 

Este estudo contribui, especialmente, com a vigilância em saúde por identificar grupos populacionais com maior vulnerabilidade à COVID Longa, em especial pessoas idosas, mulheres e pessoas não vacinadas. Os achados destacam a importância do acompanhamento contínuo de pacientes infectados pela COVID-19 na APS, além de oferecerem subsídios para uma vigilância em saúde mais direcionada e efetiva. Além disso, a presente análise se destaca na abordagem simultânea dos fatores sexo, faixa etária e esquema vacinal como preditores da manifestação de sintomas prolongados da COVID-19 em uma população assistida no contexto de um sistema público de saúde. Ainda, o uso de dados oriundos de sistemas oficiais confere robustez metodológica à análise e amplia o potencial de aplicabilidade dos resultados para a gestão em saúde pública. 

Deste modo, os resultados obtidos podem oferecer subsídios importantes para a prática clínica ao evidenciar os fatores de risco para a COVID Longa, contribuindo diretamente na qualificação dos serviços de saúde - em especial da APS e da vigilância em saúde - por meio da formulação de protocolos clínicos direcionados à detecção precoce dos sintomas e à estratificação de risco com base em variáveis sociodemográficas e clínicas. Tais medidas contribuem para um cuidado mais resolutivo, fortalecimento da vigilância e melhor uso dos recursos disponíveis.

No âmbito da enfermagem, os achados reforçam a importância da atuação estratégica dos profissionais na vigilância clínica, acolhimento e no acompanhamento contínuo de usuários que apresentam sintomas persistentes após a infecção pela COVID-19. As evidências do estudo apoiam práticas como escuta qualificada e planejamento do cuidado, permitindo uma abordagem mais proativa e individualizada. O reconhecimento dos sintomas mais frequentes, aliado à valorização do relato subjetivo e à consideração de fatores como sexo, idade e vacinação, contribui para um cuidado mais humanizado, resolutivo e alinhado às evidências científicas.

Apesar das contribuições relevantes, este estudo apresenta algumas limitações que devem ser consideradas na interpretação dos resultados. A principal limitação refere-se à dependência de dados secundários provenientes de prontuários eletrônicos, os quais podem conter registros incompletos ou inconsistentes. Essa limitação pode ter levado à subnotificação de sintomas ou à omissão de informações clínicas relevantes, comprometendo a acurácia na identificação de casos de COVID Longa. Outra limitação da pesquisa diz respeito ao tamanho da amostra, que não captura a diversidade da população brasileira. Além disso, o delineamento transversal adotado não permite estabelecer relações de causalidade entre os fatores analisados e o desenvolvimento da COVID Longa, restringindo a análise a associações estatísticas. 

O uso de informações de apenas um município também limita a generalização dos achados para outras regiões, especialmente considerando as desigualdades regionais no acesso à saúde, estrutura da APS e cobertura vacinal no Brasil. Adicionalmente, a definição de COVID Longa utilizada, embora baseada na proposta da OMS, depende da presença de sintomas autorreferidos e do retorno do paciente ao serviço de saúde após a fase aguda, o que pode excluir indivíduos que, mesmo com sintomas persistentes, não buscaram atendimento. 

## Conclusão

Este estudo evidenciou que pessoas idosas apresentaram maior prevalência de falta de ar; pessoas do sexo feminino apresentaram maior prevalência de queda de cabelo como sintoma de COVID Longa; e indivíduos não vacinados apresentaram maior prevalência de dor no peito. Determinar os sintomas de COVID Longa mais prevalentes, considerando a faixa etária, sexo e situação vacinal, pode contribuir na avaliação clínica da doença, bem como na identificação de possíveis fatores considerados de risco para alguns sintomas manifestados na doença, auxiliando na elaboração de protocolos que orientem o diagnóstico e manejo clínico da doença. 

Os achados também ressaltam a importância da vacinação contra a COVID-19 como medida preventiva não apenas contra formas graves da doença aguda, mas também contra manifestações prolongadas. Nesse sentido, estratégias de ampliação da cobertura vacinal e de reforço às campanhas públicas se mostram fundamentais para reduzir o impacto da COVID Longa na população. 

Por fim, embora o estudo tenha sido realizado em um único município, seus resultados apresentam aplicabilidade a contextos semelhantes, como cidades de médio porte com organização assistencial comparável. Ainda assim, recomenda-se a realização de estudos multicêntricos, métodos longitudinais e a incorporação de variáveis sociais e comportamentais, amostras representativas de diferentes regiões do país, a fim de validar os achados e aprofundar o conhecimento sobre os fatores associados à COVID Longa no contexto brasileiro.

## Data Availability

Os conjuntos de dados relacionados a este artigo estarão disponíveis mediante solicitação ao autor correspondente.

# Translation: Predictores de COVID prolongada: ancianos, mujeres y vacunación incompleta contra la COVID-19^*^

**DOI:** 10.1590/1518-8345.7954.4815

**Authors:** Karina Marques Prediger, Ana Cristina Ribeiro La Scaléa, Silvia Carla da Silva André Uehara

**Affiliations:** 1 Universidade Federal de São Carlos, Departamento de Enfermagem, São Carlos, SP, Brasil.; 2 Becaria de la Fundação de Amparo à Pesquisa do Estado de São Paulo (FAPESP), Brasil.; 3 Becaria de la Coordenação de Aperfeiçoamento de Pessoal de Nível Superior (CAPES), Brasil.

**Keywords:** Síndrome Post Agudo de COVID-19, Prevalencia, Femenino, Factores de Edad, Vacunas, Signos y Síntomas.

## Abstract

**Objetivo:**

analizar los síntomas de COVID prolongada en pacientes atendidos en Atención Primaria de Salud, considerando sexo, edad y esquema de vacunación.

**Método:**

estudio transversal y analítico, realizado con una muestra de 350 pacientes con COVID prolongada, atendidos en servicios de Atención Primaria de Salud. Los datos se recolectaron a través del análisis de historias clínicas y se analizaron mediante el modelo de regresión de Poisson.

**Resultados:**

los ancianos tuvieron una prevalencia 88% mayor de dificultad para respirar como síntoma de COVID prolongada; las mujeres tenían una prevalencia 13,5 veces mayor de pérdida de cabello; Además, las personas no vacunadas tenían una prevalencia 3.64 veces mayor de reportar dolor en el pecho.

**Conclusión:**

la identificación de los síntomas más prevalentes de COVID prolongada, considerando el grupo de edad, el sexo y el estado de vacunación, puede contribuir a la evaluación clínica y al desarrollo de protocolos que orienten el manejo adecuado de estas personas.

## Destacados

**(1)** Los ancianos presentaron una mayor prevalencia de dificultad respiratoria en la CP. **(2)** Las mujeres presentaron una mayor prevalencia de caída del cabello en la CP. **(3)** Las personas no vacunadas presentaron una mayor prevalencia de dolor torácico en la CP.

## Introducción

La COVID prolongada se caracteriza por antecedentes de infección por COVID-19, cuyos síntomas persisten o fluctúan durante al menos dos meses después de la fase aguda y que no pueden justificarse con un diagnóstico alternativo. Entre los síntomas más frecuentes de COVID prolongada, se destacan la fatiga, la dificultad para respirar, la disfunción cognitiva y otros que impactan en las actividades diarias del paciente^1^.

Se estima que la prevalencia global de COVID prolongada es de aproximadamente 36% y que la prevalencia varía según la región geográfica, habiéndose estimado en 35% en Asia, 39% en Europa, 30% en América del Norte y 51% en América del Sur^2^. En Brasil, también hay una variación regional en la prevalencia de COVID prolongada. Entre marzo de 2020 y marzo de 2023, los estados con mayores coeficientes por cada 100 mil habitantes fueron Goiás, Distrito Federal, Paraná, Rio Grande do Sul, Tocantins y São Paulo^3^.

En el período de septiembre de 2021 a diciembre de 2024, se registraron 167.138 consultas individuales en Atención Primaria de Salud (APS) como condición de salud no especificada después de COVID-19 (CIE-U09.9). El estado de São Paulo tuvo la mayor incidencia acumulada de atención post-COVID en el país, con 2.026,9 visitas por cada 100 mil habitantes, seguido de los estados de Amazonas, Rio Grande do Sul, Minas Gerais y Tocantins^3^.

Así, aunque los coeficientes de COVID prolongada varían a nivel mundial y nacional, es posible observar que una parte de los pacientes infectados con COVID-19 puede desarrollar síntomas prolongados^4^. Así, la COVID prolongada es una condición que abarca manifestaciones heterogéneas y multisistémicas, que involucran diferentes sistemas de órganos, como el cardiovascular, gastrointestinal, endocrino y neuropsiquiátrico^5^ ^-^ ^7^. Así, el profesional de la salud que trata a un paciente con estos síntomas puede relacionarlos fácilmente con causas psicosomáticas, como depresión y ansiedad, lo que puede dificultar el diagnóstico de COVID prolongada^4^.

Cabe señalar que los pacientes que desarrollaron la forma grave de COVID-19 tienden a manifestar síntomas prolongados después de la fase aguda de la enfermedad. En este contexto, se verificó la prevalencia de síntomas persistentes en pacientes atendidos de forma ambulatoria, con 5% a 26% de estas personas presentando síntomas prolongados de COVID-19 por un período de uno a tres meses, y de 2% a 62% presentando síntomas por un período de tres a seis meses después del diagnóstico de la enfermedad aguda^8^.

La fisiopatología de la COVID prolongada puede estar relacionada con diferentes mecanismos, según el sistema de órganos afectado. Así, el desarrollo de COVID prolongada implica secuelas continuas de tejidos gravemente dañados, persistencia del SARS-CoV-2 en órganos, desregulación del sistema inmunológico que desencadena autoinmunidad e inflamación crónica, hipoxia tisular y daño endotelial^5^.

A pesar de que la literatura presenta la descripción de los síntomas manifestados en la COVID prolongada^1^ ^,^ ^4^, dada la similitud con otras enfermedades, caracterizar el cuadro sintomatológico más prevalente, según el sexo, el grupo de edad y el estado de vacunación contra la COVID-19, puede ayudar a identificar el grupo poblacional con mayor probabilidad de desarrollar esta condición. Así, este estudio tiene como objetivo analizar los síntomas de COVID prolongada en pacientes atendidos en Atención Primaria de Salud, considerando sexo, edad y calendario de vacunación.

## Método

### Tipo de estudio

Se trata de un estudio transversal y analítico, desarrollado de acuerdo con las recomendaciones del *Strengthening The Reporting of Observational Studies in Epidemiology* (STROBE)^9^.

### Escenario

Este estudio se realizó en la ciudad de São Carlos, ubicada en el estado de São Paulo. Los datos se obtuvieron de los servicios de Vigilancia de la Salud y del Departamento de Gestión y Atención Ambulatoria, sector responsable de los servicios de Atención Primaria de Salud (APS).

### Periodo

Para el cálculo de la muestra se utilizaron datos referentes a casos de COVID-19 en la población adulta reportados en el período del 26/02/2020 al 26/02/2024. Así, fue posible identificar, en este intervalo, el diagnóstico CIE-Z20 (COVID-19 confirmado) y, al menos, un retorno al servicio de APS dos meses después de la fecha de infección aguda por COVID-19. La recopilación de datos se realizó entre agosto de 2023 y junio de 2024.

### Población y muestra del estudio

Se realizó un cálculo del tamaño muestral considerando todos los casos de COVID-19 diagnosticados en la población mayor de 18 años del municipio, referidos al período comprendido entre el 26/02/2020 y el 26/02/2024, utilizando Prueba Rápida (TR) o Reacción en Cadena de la Polimerasa con Transcriptasa Inversa (RT-PCR). En la ciudad de São Carlos-SP, se notificaron 62.977 casos positivos de COVID-19 en personas mayores de 18 años durante el período de análisis de este estudio. La elección de la prevalencia se basó en datos de la literatura^10^, que indican que cerca de 50% de los casos de COVID-19 tenían síntomas de COVID prolongada.

Para el cálculo de la muestra, se asumió un nivel de significancia del 5% (α = 0,05), un error absoluto tolerable de 5 puntos porcentuales (d = 0,05) y una población finita estimada en N = 31.489 pacientes con COVID prolongada mayores de 18 años en el municipio de interés. La prevalencia esperada utilizada fue p = 0,356, correspondiente a la frecuencia del síntoma fatiga, una de las manifestaciones más reportadas en estudios previos sobre COVID prolongada. Por lo tanto, el tamaño de la muestra adoptado en este estudio fue n = 350. El cálculo se realizó mediante la siguiente ecuación:

$$n=\frac{z_{\left(1-\frac{\alpha }{2}\right)}^{2}Np\left(1-p\right)}{d^{2}\left(N-1\right)+z_{\left(1-\frac{\alpha }{2}\right)}^{2}p\left(1-p\right)}$$

Donde p es la prevalencia esperada, d es el error absoluto tolerable, N es el tamaño de la población y z es el valor de la distribución normal estándar correspondiente al nivel de confianza deseado.

### Criterios de selección

Se incluyeron en el estudio pacientes adultos mayores de 18 años que dieron positivo para COVID-19 y regresaron a los servicios de APS con síntomas de COVID prolongada, según la definición de la OMS, que describe los síntomas como recidivantes o fluctuantes durante un período mínimo de dos meses después de la infección con la enfermedad aguda^1^. Se excluyeron del estudio los pacientes cuyas historias clínicas no eran accesibles, que no vivían en lugares asignados a la APS o que fallecieron.

### Variables del estudio

Las variables del estudio fueron sexo, edad, raza/color, comorbilidades, esquema de vacunación y síntomas reportados. Entre los síntomas reportados se encuentran cambios en la menstruación, ansiedad, cansancio, secreción nasal, diarrea, dificultad para concentrarse, dolor: abdominal, articular, cabeza, garganta, miembros inferiores (MMII), miembros superiores (MMSS), musculares, espalda/lumbares, oído, pecho, dificultad para respirar, fiebre, hormigueo en MMII y/o MMSS. Además, hay debilidad muscular/pérdida de fuerza, insomnio, náuseas, odinofagia, pérdida de memoria/confusión, pérdida del olfato, pérdida del gusto, pérdida de cabello, queja dental de pérdida de dientes, mareos, tos, tristeza/depresión y vómitos. Este estudio consideró síntomas autoreferidos compatibles con la definición de COVID prolongada de la OMS^1^, es decir, generalmente con inicio tres meses después de la fase aguda de la infección, con una duración de al menos dos meses y sin otra explicación clínica identificable.

### Instrumentos utilizados para la recopilación de datos de información

Para la recopilación de datos, se construyeron hojas de cálculo de *Microsoft Excel*, que contenían las variables de interés. Inicialmente, se crearon diferentes pestañas para recopilar datos de las diferentes bases de datos utilizadas en esta investigación, obtenidas de SisCOVID, el sistema de notificación de COVID-19 del municipio de São Carlos-SP. En la siguiente pestaña, se ingresaron datos sobre el estado de vacunación, obtenidos de VaciVida, un sistema desarrollado por el Estado de São Paulo. Finalmente, en la última pestaña, se insertaron los datos relativos a los síntomas de COVID prolongada obtenidos del Registro Ciudadano Electrónico (PEC). Se adoptaron medidas para minimizar los sesgos, incluida la capacitación de los recolectores de datos - dos estudiantes de pregrado, dos estudiantes de maestría y un estudiante de doctorado en Ciencias de la Salud -, el uso de criterios de inclusión y exclusión previamente definidos, y la estandarización de la recolección de datos a través de una hoja de cálculo estructurada y digitalizada. Además, se destaca la elección del nivel de significancia adecuado (α = 0,05), el ajuste por comparaciones múltiples, la aplicación de métodos estadísticos apropiados y el tamaño muestral robusto.

### Recopilación de datos

En primer lugar, se realizó la identificación de casos confirmados de COVID-19 en SisCOVID; posteriormente, se consultó a la PEC para ver si el paciente infectado con SARS-CoV-2 regresaba a la APS con un informe de síntomas de COVID prolongada, según la definición de la OMS^1^. A través de SisCOVID, también fue posible obtener información sobre consultas de usuarios con quejas de síntomas respiratorios después de la fase aguda de la enfermedad; por otro lado, se verificaron los síntomas de COVID prolongada reportados por los usuarios, que afectaron los diferentes sistemas de órganos del cuerpo. Además, los datos sobre vacunación se obtuvieron de la página VaciVida, de la vigilancia epidemiológica municipal.

### Tratamiento y análisis de datos

Inicialmente, los datos se describieron por medio de frecuencias absolutas y porcentuales (variables cualitativas) y por medidas de centralidad y dispersión, como media, desviación estándar, mínimo, mediana y máximo (variables cuantitativas). Para estimar la razón de prevalencia bruta de los síntomas de COVID prolongada en relación con las variables de interés, se utilizó el modelo de regresión de Poisson con varianza robusta^11^. En los análisis en los que la frecuencia de síntomas fue nula, se realizó la prueba exacta de Fisher. El uso de Datos Faltantes se indicó en la parte inferior de las tablas para garantizar la transparencia de los datos presentados, manteniendo el número de casos faltantes observados, sin imputación. Todos los análisis se realizaron con la ayuda del software SAS 9.4^12^, adoptando un nivel de significancia del 5%.

### Aspectos éticos

De acuerdo con la Resolución n.º 510/2016, el proyecto fue aprobado por el Comité de Ética en Investigación de la Universidad Federal de São Carlos (CAAE: 67128023.4.0000.5504; CAAE: 74911623.5.0000.5504).

## Resultados

Se analizaron un total de 3.778 casos notificados y confirmados de COVID-19 para llegar a una muestra de 350 casos de COVID prolongada, lo que corresponde al 12% de los casos de la enfermedad aguda en la población mayor de 18 años en el municipio de São Carlos-SP. La muestra del estudio está compuesta por 67,1% (235) mujeres y 32,9% (115) hombres; El 61,6% (215) se declararon blancos, el 30,1% (105) morenos, el 8,0% (28) negros, el 0,3% (1) amarillos y 1 caso no tenía la raza/color registrado. En cuanto al grupo de edad, el 77,1% (270) eran personas menores de 60 años y el 22,9% (80) eran personas de 60 años o más. La edad media fue de 46,9 años (Tabla 1).

Al comparar los síntomas de COVID prolongada presentados por los pacientes, considerando el sexo, se destaca que las mujeres tuvieron una prevalencia de pérdida de cabello 13,5 veces mayor en comparación con los hombres (Tabla 2).

**Tabla 1:** Caracterización demográfica y clínica de la muestra del estudio. São Carlos, SP, Brasil 2024

Características	Frecuencia (n = 350)	Porcentaje (%)
**Sexo**		
Femenino	235	67,1
Masculino	115	32,9
**Edad**		
Promedio	46,9 [15,8]	-
Mediana (Mín* e Máx^†^)	47,0 [14,0 e 88,0]	-
**Grupo de edad**		
< 60 años	270	77,1%
≥ 60 años	80	22,9%
**Raza/Color**		
Amarillo	1	0,3
Blanco	215	61,6
Marrón	105	30,1
Negro	28	8,0
*Missing* **(Ausente)**	1	-
**Comorbilidades**		
Hemoglobinopatías	1	0,3
Cirrosis hepática	0	0
Enfermedades cardiovasculares	17	4,9
Enfermedades neurológicas	4	1,1
Diabetes Mellitus (Tipo 1 y Tipo 2)	53	15,1
Enfermedad Renal Crónica	4	1,1
Hipertensión Arterial Sistémica	106	30,3
Inmunodeprimidos	0	0
Obesidad	56	16
Enfermedades pulmonares crónicas graves	21	6,0
Otros	51	14,6

*Mín. = Mínimo; ^†^Máx. = Máximo

**Tabla 2:** Comparación de la presencia de síntomas de COVID prolongada, considerando el sexo de la población de estudio

Síntomas de COVID prolongada	F* (n = 235)	M^†^ (n = 115)	Razón de prevalencia (F* *vs* M ^†^) **[IC 95%]** ^‡^	Valor p^§^
Modificación en la menstruación^||^	8 (3,6%)	0 (0%)	-	0,06^¶^
Ansiedad^||^	14 (6,3%)	7 (6,7%)	0,94 (0,39; 2,25)	0,89
Cansancio	44 (18,7%)	13 (11,3%)	1,66 (0,93; 2,95)	0,09
Rinorrea	32 (13,7%)	14 (12,2%)	1,12 (0,62; 2,02)	0,70
Diarrea	4 (1,7%)	0 (0%)	-	0,31^¶^
Dificultad para concentrarse^||^	5 (2,2%)	0 (0%)	-	0,18^¶^
Dolor abdominal^||^	13 (58%)	8 (7,6%)	0,76 (0,33; 1,78)	0,53
Dolor articular^||^	4 (1,8%)	3 (2,9%)	0,63 (0,14; 2,74)	0,53
Dolor de cabeza	72 (30,6%)	25 (21,7%)	1,41 (0,95; 2,1)	0,09
Dolor de garganta	46 (19,6%)	20 (17,4%)	1,13 (0,7; 1,81)	0,63
Dolor em miembros inferiores^||^	24 (10,7%)	15 (14,3%)	0,75 (0,41; 1,37)	0,35
Dolor em miembros superiores^||^	9 (4,0%)	6 (5,7%)	0,7 (0,26; 1,92)	0,49
Dolor muscular	37 (15,7%)	14 (12,2%)	1,29 (0,73; 2,29)	0,38
Dolor de espalda/lumbar^||^	22 (9,8%)	8 (7,6%)	1,29 (0,59; 2,8)	0,52
Dolor en el oído^||^	1 (0,4%)	0 (0%)	-	0,99^¶^
Dolor en el pecho^||^	10 (4,5%)	7 (6,7)	0,67 (0,26; 1,71)	0,40
Dolor en la caja torácica^||^	10 (4,5%)	3 (2,9%)	1,56 (0,44; 5,56)	0,49
Dificultad para respirar	33 (14,0%)	23 (20,0%)	0,7 (0,43; 1,14)	0,15
Fiebre	26 (11,1%)	8 (7,0%)	1,59 (0,74; 3,4)	0,23
Hormigueo en los miembros inferiores^||^	11 (4,9%)	5 (4,8%)	1,03 (0,37; 2,89)	0,95
Hormigueo en los miembros superiores^||^	10 (4,5%)	1 (1,0%)	4,69 (0,61; 36,14)	0,14
Debilidad/pérdida de fuerza muscular^||^	21 (9,4%)	9 (8,6%)	1,09 (0,52; 2,31)	0,81
Insomnio^||^	15 (6,7%)	4 (3,8%)	1,76 (0,6; 5,17)	0,31
Náusea	7 (3,0%)	1 (0,9%)	3,43 (0,43; 27,51)	0,25
Odinofagia^||^	1 (0,4%)	1 (1,0%)	0,47 (0,03; 7,42)	0,59
Pérdida de memoria/confusión^||^	10 (4,5%)	5 (4,8%)	0,94 (0,33; 2,67)	0,90
Pérdida de olfato	3 (1,3%)	4 (3,5%)	0,37 (0,08; 1,61)	0,18
Pérdida del gusto	3 (1,3%)	1 (0,9%)	1,47 (0,15; 13,96)	0,74
Pérdida de cabello^||^	29 (12,9%)	1 (1,0%)	13,59 (1,88; 98,45)	0,01
Queja dental de pérdida dental^||^	2 (0,9%)	1 (1,0%)	0,94 (0,09; 10,22)	0,96
Vértigo	11 (4,9%)	4 (3,8%)	1,29 (0,42; 3,95)	0,66
Tos	59 (25,1%)	27 (23,5%)	1,07 (0,72; 1,59)	0,74
Tristeza/depresión^||^	11 (4,9%)	4 (3,8%)	1,29 (0,42; 3,95)	0,66
Vómitos	9 (3,8%)	3 (2,6%)	1,47 (0,41; 5,32)	0,56

*F = Sexo femenino; ^†^M = Sexo masculino; ^‡^IC = Intervalo de Confianza; ^§^Valor p = Nivel de significación; ^||^ *Missing* (Ausentes) 20; ^¶^Valor referente a la prueba exacta de Fisher

Al comparar los síntomas de COVID prolongada de los pacientes, considerando el grupo de edad, se destaca que las personas de 60 años o más tuvieron una prevalencia 88% mayor de dificultad para respirar, además de una disminución en la prevalencia de dolor de cabeza, 66% de dolor de garganta y 63% de dolor muscular en comparación con los adultos más jóvenes (Tabla 3).

Los resultados mostraron que las personas con COVID prolongada que no recibieron ninguna dosis de vacuna contra COVID-19 tenían una prevalencia 3,64 veces mayor de dolor torácico (Tabla 4).

Los resultados muestran que el 27,2% (96) de las personas con COVID prolongada habían recibido 2 dosis de la vacuna contra la COVID-19, siendo la cefalea el síntoma principal en este grupo, presente en el 31,3% (30) de los casos analizados. Se encuentra que, independientemente de la cantidad de dosis administradas contra COVID-19, los síntomas comunes de COVID prolongada entre las personas fueron dolor de cabeza, dolor de garganta y tos (Tabla 5).

**Tabla 3:** Comparación de la presencia de síntomas de COVID prolongada, considerando el grupo de edad de la población estudiada.

Descripción de los síntomas de COVID prolongada	< 60 años (n = 270)	≥ 60 años (n = 80)	Razón de prevalencia (≥ 60 *vs* < 60) [IC 95%]*	Valor p^†^
Modificación en la menstruación^‡^	8 (3,2%)	0 (0%)	-	0,21^§^
Ansiedad^‡^	19 (7,6%)	2 (2,6%)	0,34 (0,08; 1,42)	0,14
Cansancio	47 (17,4%)	10 (12,5%)	0,72 (0,38; 1,36)	0,92
Rinorrea	41 (15,2%)	5 (6,3%)	0,41 (0,17; 1)	0,05
Diarrea	3 (1,1%)	1 (1,3%)	1,13 (0,12; 10,67)	0,92
Dificultad para concentrarse^‡^	5 (2,0%)	0 (0%)	-	0,60^§^
Dolor abdominal^‡^	16 (6,4%)	5 (6,4%)	1,01 (0,38; 2,66)	0,99
Dolor articular^‡^	5 (2,0%)	2 (2,6%)	1,29 (0,25; 6,5)	0,76
Dolor de cabeza	85 (31,5%)	12 (15,0%)	0,48 (0,27; 0,83)	<0,01
Dolor de garganta	60 (22,2%)	6 (7,5%)	0,34 (0,15; 0,89)	0,03
Dolor en miembros inferiores^‡^	29 (11,6%)	10 (12,8%)	1,11 (0,57; 2,17)	0,76
Dolor en miembros superiores^‡^	11 (4,4%)	4 (5,1%)	1,17 (0,38; 3,57)	0,78
Dolor muscular	46 (17,0%)	5 (6,3%)	0,37 (0,15; 0,89)	0,03
Dolor de espalda/espalda baja^‡^	23 (9,2%)	7 (9,0%)	0,98 (0,44; 2,19)	0,96
Dolor de oído^‡^	0 (0%)	1 (1,3%)	-	0,24^§^
Dolor en el pecho^‡^	15 (6,0%)	2 (2,6%)	0,43 (0,1; 1,84)	0,25
Dolor en la caja torácica^‡^	12 (4,8%)	1 (1,3%)	0,27 (0,04; 2,03)	0,20
Dificultad para respirar	36 (13,3%)	20 (25,0%)	1,88 (1,15; 3,05)	0,01
Hormigueo/Palpitaciones en las extremidades inferiores^‡^	14 (5,6%)	2 (2,6%)	0,46 (0,11; 1,98)	0,30
Hormigueo/Palpitaciones en las extremidades superiores^‡^	8 (3,2%)	3 (3,8%)	1,21 (0,33; 4,44)	0,78
Debilidad/pérdida de fuerza muscular^‡^	20 (8,0%)	10 (12,8%)	1,61 (0,79; 3,29)	0,19
Insomnio^‡^	15 (6,0%)	4 (5,1%)	0,86 (0,29; 2,51)	0,78
Fiebre	28 (10,4%)	6 (7,5%)	0,72 (0,31; 1,68)	0,45
Náuseas	8 (3,0%)	0 (0%)	-	0,21
Odinofagia^‡^	2 (0,8%)	0 (0%)	-	0,99^§^
Pérdida de memoria/confusión^‡^	10 (4,0%)	5 (6,4%)	1,61 (0,57; 4,57)	0,37
Pérdida de olfato	5 (1,9%)	2 (2,55)	1,35 (0,27; 6,83)	0,72
Pérdida de gusto	3 (1,1%)	1 (1,3%)	1,13 (0,12; 10,67)	0,92
Pérdida de cabello^‡^	27 (10,8%)	3 (3,8%)	0,36 (011; 1,15)	0,08
Queja dental de pérdida dental post-COVID^‡^	2 (0,8%)	1 (1,3%)	1,61 (0,15; 17,51)	0,70
Tristeza/depresión^‡^	11 (4,4%)	4 (5,1%)	1,17 (0,38; 3,57)	0,78
Vértigo^‡^	11 (4,4%)	4 (5,1%)	1,17 (0,38; 3,57)	0,78
Tos	73 (27,0%)	13 (16,3%)	0,6 (0,35; 1,03)	0,06
Vómito	10 (3,7%)	2 (2,5%)	0,68 (0,15; 3,02)	0,61

*IC = Intervalo de Confianza; ^†^Valor p = Nivel de significación; ^‡^ *Missing* (Ausentes) 21; ^§^Valor referente a la prueba exacta de Fisher

**Tabla 4:** Comparación de la presencia de síntomas de COVID prolongada, considerando el calendario de vacunación de la población de estudio. São Carlos, SP, Brasil, 2024

Descripción de los síntomas de COVID prolongada	Antes de la vacuna (n = 295)	Después de la vacuna (n = 47)	Razón de prevalencia [IC 95%]*	Valor p^†^
Alteración en la menstruación^‡^	6 (2,1%)	2 (4,8%)	2,22 (0,46; 10,65)	0,32
Ansiedad^‡^	18 (6,4%)	3 (7,1%)	1,11 (0,34; 3,61)	0,86
Cansancio	53 (18,0%)	4 (8,5%)	0,47 (0,18; 1,25)	0,13
Rinorrea	36 (12,2%)	10 (21,3%)	1,74 (0,93; 3,26)	0,09
Diarrea	4 (1,4%)	0 (0%)	-	0,99^§^
Dificultad para concentrarse^‡^	4 (1,4%)	1 (2,4%)	1,67 (0,19; 14,56)	0,64
Dolor abdominal^‡^	19 (6,8%)	2 (4,8%)	0,7 (0,17; 2,9)	0,63
Dolor articular^‡^	6 (2,1%)	1 (2,4%)	1,11 (0,14; 9)	0,92
Dolor de cabeza	83 (28,1%)	13 (27,7%)	0,98 (0,6; 1,62)	0,95
Dolor de garganta	53 (18,0%)	12 (25,5%)	1,42 (0,82; 2,45)	0,21
Dolor en miembros inferiores^‡^	36 (12,9%)	2 (4,8%)	0,37 (0,09; 1,48)	0,16
Dolor en miembros superiores	14 (5,0%)	1 (2,4%)	0,48 (0,06; 3,53)	0,47
Dolor muscular	45 (15,3%)	6 (12,8%)	0,84 (0,38; 1,85)	0,66
Dolor de espalda/espalda baja/lumbar^‡^	26 (9,3%)	3 (7,1%)	0,77 (0,24; 2,43)	0,65
Dolor de oído^‡^	1 (0,4%)	0 (0%)	-	0,99^§^
Dolor en el pecho	11 (3,9%)	6 (14,3%)	3,64 (1,42; 9,31)	<0,01
Dolor en la caja torácica^‡^	10 (3,6%)	3 (7,1%)	2 (0,57; 6,97)	0,28
Dificultad para respirar	48 (16,3%)	7 (14,9%)	0,92 (0,44; 1,9)	0,81
Hormigueo/palpitaciones en miembros inferiores^‡^	11 (3,9%)	4 (9,5%)	2,42 (0,81; 7,26)	0,11
Hormigueo/palpitaciones en miembros superiores^‡^	10 (3,6%)	1 (2,4%)	0,67 (0,09; 5,08)	0,70
Debilidad/pérdida de la fuerza muscular^‡^	27 (9,6%)	3 (7,1%)	0,74 (0,24; 2,33)	0,61
Insomnio^‡^	15 (5,4%)	3 (7,1%)	1,33 (0,4; 4,41)	0,64
Fiebre	30 (10,2%)	4 (8,5%)	0,84 (0,31; 2,27)	0,73
Náusea	7 (2,4%)	1 (2,1%)	0,9 (0,11; 7,12)	0,92
Odinofagia^‡^	1 (0,4%)	1 (2,4%)	6,67 (0,43; 104,57)	0,18
Pérdida de memoria/confusión^‡^	13 (4,6%)	1 (2,4%)	0,51 (0,07; 3,82)	0,51
Pérdida de olfato	5 (1,7%)	2 (4,3%)	2,51 (0,5; 12,57)	0,26
Pérdida de gusto	3 (1,0%)	1 (2,1%)	2,09 (0,22; 19,69)	0,52
Pérdida de cabello^‡^	26 (9,3%)	4 (9,5%)	1,03 (0,38; 2,79)	0,96
Queja dental de pérdida dental post-COVID^‡^	3 (1,11%)	0 (0%)	-	0,99^§^
Tristeza/depresión^‡^	12 (4,3%)	2 (4,8%)	1,11 (0,26; 4,79)	0,89
Vértigo^‡^	12 (4,3%)	3 (7,1%)	1,67 (0,49; 5,66)	0,41
Tos	74 (25,1%)	11 (23,4%)	0,93 (0,54; 1,62)	0,81
Vómito	10 (3,4%)	2 (4,3%)	1,26 (0,28; 5,55)	0,76

*IC = Intervalo de Confianza; ^†^Valor p = Nivel de significación; ^‡^ *Missing* (Ausentes) 21; ^§^Valor referente a la prueba exacta de Fisher

**Tabla 5:** Descripción de los síntomas de COVID prolongada, considerando las dosis administradas en el esquema de vacunación de los pacientes. São Carlos, SP, Brasil, 2024

Síntomas de COVID prolongada	Dosis de vacuna administradas contra el COVID-19					
	0 (n = 47)	1 (n = 47)	2 (n = 96)	3 (n = 86)	4 (n = 53)	5 (n = 13)
Alteración en la menstruación*	2 (4,8%)	1 (2,4%)	4 (4,4%)	0 (0%)	1 (1,9%)	0 (0%)
Ansiedad*	3 (7,1%)	6 (14,3%)	6 (6,6%)	5 (6,0%)	1 (1,9%)	0 (0%)
Cansancio	4 (8,5%)	9 (19,1%)	16 (16,7%)	18 (20,9%)	9 (17,0%)	1 (7,7%)
Rinorrea	10 (21,3%)	5 (10,9%)	9 (9,4%)	9 (10,5%)	13 (24,5%)	0 (0%)
Diarrea	0 (0%)	0 (0%)	2 (2,1%)	2 (2,3%)	0 (0%)	0 (0%)
Dificultad para concentrarse*	1 (2,4%)	0 (0%)	3 (3,3%)	0 (0%)	1 (1,9%)	0 (0%)
Dolor abdominal*	2 (4,8%)	3 (7,1%)	3 (3,3%)	9 (10,8%)	2 (3,8%)	2 (16,7%)
Dolor articular*	1 (2,4%)	1 (2,4%)	3 (3,3%)	2 (2,4)	0 (0%)	0 (0%)
Dolor de cabeza	13 (27,7%)	14 (29,8%)	30 (31,3%)	23 (26,7%)	13 (24,5%)	3 (23,1%)
Dolor de garganta	12 (25,5%)	9 (19,1%)	16 (16,7%)	14 (16,3%)	10 (18,9%)	4 (30,8%)
Dolor en miembros inferiores*	2 (4,8%)	6 (14,3%)	10 (11,0%)	10 (12,0%)	9 (17,3%)	1 (8,3%)
Dolor en miembros superiores*	1 (2,4%)	3 (7,1%)	6 (6,6%)	2 (2,4%)	3 (5,8%)	0 (0%)
Dolor muscular	6 (12,8%)	7 (14,9%)	17 (17,7%)	11 (12,8%)	9 (17,0%)	1 (7,7%)
Dolor de espalda/espalda baja/lumbar*	3 (7,1%)	5 (11,9%)	6 (6,6%)	7 (8,4%)	8 (15,4%)	0 (0%)
Dolor de oído*	0 (0%)	0 (0%)	1 (1,1%)	0 (0%)	0 (0%)	0 (0%)
Dor en el pecho*	6 (14,3%)	2 (4,8%)	3 (3,3%)	2 (3,8%)	2 (3,8%)	1 (8,3%)
Dolor en la caja torácica*	3 (7,1%)	2 (4,8%)	4 (4,4%)	2 (2,4%)	2 (3,8%)	0 (0%)
Dificultad para respirar	7 (14,9%)	3 (6,4%)	17 (17,7%)	17 (19,8%)	11 (20,8%)	0 (0%)
Hormigueo/palpitaciones en miembros inferiores*	4 (9,5%)	3 (7,1%)	5 (5,5%)	2 (2,4%)	0 (0%)	1 (8,3%)
Hormigueo/palpitaciones em miembros superiores*	1 (2,4%)	2 (4,8%)	4 (4,4%)	2 (2,4%)	1 (1,9%)	1 (8,3%)
Debilidad/pérdida de la fuerza muscular*	3 (7,1%)	8 (19,0%)	8 (8,8)	4 (4,8%)	7 (13,5%)	0 (0%)
Insomnio*	3 (7,1%)	3 (7,1%)	5 (5,5%)	4 (4,8%)	2 (3,8%)	1 (8,3%)
Fiebre	4 (8,5%)	5 (10,6%)	8 (8,3%)	10 (11,6%)	5 (9,4%)	2 (15,4%)
Náusea	1 (2,1%)	0 (0%)	3 (3,1%)	1 (1,2%)	3 (5,7%)	0 (0%)
Odinofagia*	1 (2,4%)	0 (0%)	0 (0%)	0 (0%)	0 (0%)	1 (8,3%)
Pérdida de memoria/confusión*	1 (2,4%)	0 (0%)	4 (4,4%)	6 (7,2%)	1 (1,9%)	2 (16,7%)
Pérdida de olfato	2 (4,3%)	1 (2,1%)	1 (1,0%)	2 (2,3%)	0 (0%)	1 (7,7%)
Pérdida de gusto	1 (2,1%)	1 (2,1%)	0 (0%)	1 (1,2%)	0 (0%)	1 (7,7%)
Pérdida de cabello*	4 (9,5%)	4 (9,5%)	8 (8,8%)	7 (8,4%)	6 (11,5%)	1 (8,3%)
Queja dental de pérdida dental post-COVID*	0 (0%)	0 (0%)	1 (1,1%)	2 (2,4%)	0 (0%)	0 (0%)
Tristeza/depresión*	2 (4,8%)	3 (7,1%)	3 (3,3%)	3 (3,6%)	3 (5,8%)	0 (0%)
Vértigo*	3 (7,1%)	2 (4,8%)	5 (5,5%)	4 (4,8%)	1 (1,9%)	0 (0%)
Tos	11 (23,4%)	12 (25,5%)	26 (27,1%)	20 (23,3%)	12 (22,6%)	4 (30,8%)
Vómito	2 (4,3%)	0 (0%)	5 (5,2%)	2 (2,3%)	2 (3,8%)	1 (7,7%)
Otros^†^	8 (19,0%)	10 (23,8%)	22 (24,4%)	18 (22,0%)	16 (31,4%)	4 (33,3%)

*Missing (Ausentes) 20; ^†^ *Missing* (Ausentes) 23

## Discusión

Este estudio mostró una mayor prevalencia de dificultad para respirar entre las personas de 60 años o más; mayor prevalencia de pérdida de cabello como síntoma de COVID prolongada entre las mujeres; y mayor prevalencia de dolor torácico entre las personas no vacunadas.

Aunque la Organización Mundial de la Salud (OMS) declaró el fin de la emergencia sanitaria mundial causada por COVID-19 en mayo de 2023, el virus continúa propagándose por todo el mundo. Además del riesgo de nuevos brotes, las personas infectadas por SARS-CoV-2 representan un desafío para la salud pública mundial, debido a las manifestaciones sintomáticas que persisten o aparecen después de la fase aguda de la infección^13^.

Los mecanismos fisiopatológicos de la COVID prolongada aún se están aclarando y la evidencia indica que la afección puede deberse a múltiples factores. Entre las principales hipótesis para su patogénesis, se destacan: desregulación inmunológica con posible persistencia de reservorios virales en los tejidos, desregulación metabólica, impactos de la infección en la microbiota, disfunción endotelial, procesos autoinmunes y síndrome de tratamiento post-intensivo^14^ ^-^ ^15^.

Se ha observado que el ácido ribonucleico (ARN) del SARS-CoV-2 puede persistir durante semanas en pacientes clínicamente recuperados de COVID-19, detectándose en el tracto respiratorio, gastrointestinal, sanguíneo y líquido cefalorraquídeo^13^. Esta persistencia inflamatoria a largo plazo puede explicarse por la presencia de antígenos virales residuales, lo que provoca la activación persistente de las células T, proceso que puede durar hasta 6 meses después de la infección aguda^16^.

Concomitantemente, de acuerdo con el perfil inmunológico de los pacientes que se recuperaron del SARS-CoV-2, fue posible evidenciar la presencia continua de inflamación, daño vascular y diferenciación de las células inmunes en el período de dos a ocho meses después de la infección. Estos pacientes presentaron niveles elevados de citocinas en relación a individuos sanos, especialmente interleucina 6 (IL-6), capaz de atravesar la barrera hematoencefálica^14^.

Además, la COVID prolongada puede estar asociado a un estado de hipersensibilidad generalizada, lo que explicaría la multiplicidad de síntomas reportados por los pacientes. Entre los síntomas más frecuentes destacan el dolor y la tos, que tienen mecanismos similares de control y sensibilización periférica en sus respectivas vías aferentes. Del mismo modo, el síndrome de fatiga crónica también se ha relacionado con alteraciones en el procesamiento del dolor y la sensibilización sensorial neurogénica, tanto periférica como central. En este contexto, se observa que las cortezas insular y cingulada, áreas involucradas en el procesamiento nociceptivo de la disnea, también son activadas por el dolor y la tos^17^.

Cabe destacar que los hallazgos de este estudio corroboran la literatura sobre los síntomas más frecuentes en la COVID prolongada, entre los que destacan la tos y los dolores - especialmente el dolor de garganta y el dolor de cabeza - como los síntomas más prevalentes, independientemente del estado de vacunación, ya que son reportados tanto por personas no vacunadas como por aquellas con el esquema de vacunación completo contra la COVID-19.

En cuanto a la fisiopatología de la cefalea en la COVID prolongada, una de las hipótesis sugiere que la invasión del SARS-CoV-2 en el sistema nervioso central afecta directamente a las vías neuronales. Los estudios han demostrado una reducción de la sustancia en la circunvolución parahipocampal y la corteza orbitofrontal en pacientes con COVID-19, además de la presencia de niveles elevados de citoquinas e interleucinas en la sangre, lo que puede promover la activación inmune persistente. Sin embargo, la cefalea prolongada observada en los casos de COVID prolongada aún representa una laguna en el conocimiento científico, lo que requiere más investigaciones para esclarecerla completamente^18^.

En cuanto a los síntomas neurootorrinolaringológicos en la COVID prolongada, como dolor de garganta y tos, la explicación puede estar relacionada con problemas de coagulación, lesión inmunomediada, hipoxia resultante de insuficiencia respiratoria y la propia invasión viral directa de los tejidos. El SARS-Cov-2 puede causar daño directo al unirse al receptor de la enzima convertidora de angiotensina 2, comúnmente expresado en las vías respiratorias superiores, incluidos los tractos nasal, laríngeo y traqueal, así como en el epitelio neuroolfativo y los pulmones^19^.

Según los resultados de este estudio, las mujeres eran más susceptibles a desarrollar COVID prolongada. Según el Centro Nacional de Estadísticas de Salud, la COVID prolongada afecta al doble de mujeres que de hombres, lo que presenta un riesgo elevado en mujeres premenopáusicas. Estas evidencias sugieren que las hormonas sexuales tienen potencial en el desarrollo de COVID prolongada^20^.

Por lo tanto, la entrada del SARS-CoV-2 puede verse facilitada por los andrógenos a través de la enzima proteasa serina transmembrana 2 (TMPRSS 2). Además, los receptores de andrógenos se encuentran en el cromosoma X, determinante del sexo biológico femenino. Así, el andrógeno circulante promueve la transcripción de la TMPRSS 2, que prepara al SARS-CoV-2 para interactuar con los receptores de la enzima convertidora de angiotensina 2 (ECA 2), permitiendo su entrada en las células huéspedes^6^ ^).^

A través de la codificación de andrógenos, se realiza una sola copia del cromosoma X, lo que permite correlacionar los diferentes niveles de sensibilidad a los andrógenos. Así, a través de estas modificaciones, existe un mayor riesgo de enfermedades que están mediadas por andrógenos, como la alopecia. Concomitantemente con el análisis de este estudio, fue posible identificar que las mujeres tenían una mayor prevalencia de pérdida de cabello. En resumen, las acciones mediadas por la enzima androgénica TMPRSS 2 pueden explicar las diferencias entre los sexos en relación a los resultados de la infección por SARS-CoV-2^6^.

En este estudio, al abordar la edad como una variable asociada a la ocurrencia de COVID prolongada, se evidenció que los ancianos tenían una mayor prevalencia de dificultad para respirar. En este contexto, la literatura ha señalado que los hallazgos clínicos y las características de la COVID prolongada pueden diferir entre los grupos de edad^21^ ^-^ ^22^. Un análisis mostró que los participantes mayores de 65 años tenían una mayor frecuencia de síntomas de COVID prolongada^21^. Aunque los síntomas de la COVID prolongada, como la fatiga y la disnea, son comunes tanto en la población adulta como en la de edad avanzada, otros síntomas, como dolor de cabeza, dolor en el pecho, palpitaciones, problemas de concentración y angustia emocional, fueron más frecuentes entre las personas más jóvenes. Por otro lado, la tos y la artralgia fueron más comunes en personas mayores^21^ ^-^ ^22^.

En este escenario, se observa que los síntomas que se manifiestan en el COVID prolongada pueden variar según el grupo de edad. Una hipótesis para esta variación está relacionada con el daño sufrido por el cuerpo, considerando la gravedad de la infección aguda por COVID-19. Otra posible explicación involucra las diferencias fisiológicas entre los grupos de edad. En este sentido, condiciones como la reserva reducida y la sarcopenia, asociadas al proceso de envejecimiento, pueden presentar diferentes efectos y consecuencias después de una infección viral, como, por ejemplo, la presencia más frecuente de síntomas como fatiga y disnea en personas mayores^21^.

Cabe destacar que muchos síntomas de COVID prolongada en ancianos pueden ser difíciles de diagnosticar, ya que pueden superponerse con otras condiciones de salud o estar asociados al proceso de envejecimiento^21^ ^-^ ^23^. Además, la diversidad de síntomas representa otro factor que dificulta el diagnóstico de la COVID prolongada. Así, ante la incertidumbre en cuanto a la atribución de síntomas a la COVID prolongada, además del relato del paciente en la evaluación clínica, puede ser necesario realizar pruebas complementarias, así como derivación a especialistas, para ayudar a definir el diagnóstico y dirigir el tratamiento adecuado^24^.

En este contexto, es importante destacar que todas las personas que han tenido la infección por COVID-19, independientemente de su gravedad, son susceptibles a la COVID prolongada, y la vacunación contra el COVID-19 es un factor fuertemente asociado a la reducción de la probabilidad de manifestar síntomas persistentes. En este estudio, se evidenció que las personas no vacunadas tenían una mayor prevalencia de tener dolor torácico como síntoma de COVID prolongada.

El síntoma de dolor torácico después de la infección por COVID-19 se relaciona comúnmente con la gravedad de la enfermedad en su fase aguda, resultante de condiciones como el uso prolongado de ventilación mecánica invasiva, síndrome de dificultad respiratoria aguda, miocarditis, pericarditis, neumonía multilobular y embolia pulmonar^25^.

En esta coyuntura, se sabe que la vacunación contra la COVID-19 es fundamental para la prevención de formas graves de infección viral en la fase aguda. La literatura indica que las personas infectadas por la enfermedad y que no fueron vacunadas tienen mayor riesgo de eventos cardiovasculares después de la infección viral^26^. Así, los síntomas torácicos de la COVID prolongada podrían explicarse como resultado de la localización de los sistemas u órganos que sufrieron daños durante la fase aguda de la enfermedad^25^ ^-^ ^26^ ^).^

En el contexto general de la manifestación de los síntomas de COVID prolongada, un análisis en el Reino Unido, Estonia y España mostró que una dosis de la vacuna COVID-19 se asoció con un riesgo reducido de desarrollar COVID prolongada^27^. Por otro lado, otro análisis mostró que las personas que recibieron una dosis de la vacuna no adquirieron protección contra la COVID prolongada, mientras que las que recibieron dos dosis la obtuvieron. Además, las personas vacunadas contra COVID-19 tenían un menor riesgo de disfunción cognitiva, problemas renales, mialgia y trastornos del sueño^28^.

En otro estudio en Brasil, se encontró que un esquema de vacunación incompleto contra COVID-19 se asoció con una mayor probabilidad de desarrollar COVID prolongada. Además, la frecuencia de síntomas como fatiga y cefaleas fue mayor entre las personas con esquemas de vacunación incompletos^29^. Cabe destacar que la vacunación redujo el riesgo de desarrollar COVID prolongada tanto en personas vacunadas antes de la infección por COVID-19 como en personas vacunadas después de la infección por la enfermedad^28^.

En este contexto, la vacunación contra COVID-19 actúa directamente sobre el aumento de los títulos de anticuerpos y la eliminación de posibles reservorios virales existentes en el cuerpo, lo que implica, en consecuencia, la reducción de la gravedad de la infección aguda por SARS-CoV-2 - un factor de riesgo conocido para el desarrollo de COVID prolongada^30^ ^-^ ^33^.

La diversidad de síntomas que se pueden atribuir a la COVID prolongada y la dificultad para definir el diagnóstico son un desafío para los servicios de salud. En este sentido, la adhesión a la vacunación contra la COVID-19 es la forma más eficaz de evitar la COVID prolongada^23^ ^,^ ^28^ ^-^ ^29^ ^,^ ^34^.

Por lo tanto, es fundamental que la evaluación clínica de los pacientes incluya el informe de síntomas subjetivos, antecedentes de infección previa por COVID-19 y la existencia de factores de riesgo que puedan influir en la manifestación de los síntomas de COVID prolongada. Así, el cuidado de la salud puede ser dirigido individualmente, y se recomienda un abordaje multidisciplinario que contemple los aspectos físicos y psicológicos de cada individuo, con el objetivo de restablecer la salud y el estado funcional de la persona^23^ ^,^ ^34^.

Este estudio contribuye, especialmente, a la vigilancia de la salud al identificar grupos poblacionales con mayor vulnerabilidad a la COVID prolongada, especialmente personas mayores, mujeres y personas no vacunadas. Los hallazgos destacan la importancia del monitoreo continuo de los pacientes infectados por COVID-19 en la APS, además de ofrecer subsidios para una vigilancia de la salud más específica y efectiva. Además, el presente análisis se destaca en el abordaje simultáneo de los factores género, grupo etario y esquema de vacunación como predictores de la manifestación de síntomas prolongados de COVID-19 en una población atendida en el contexto de un sistema público de salud. Además, el uso de datos de sistemas oficiales confiere robustez metodológica al análisis y amplía la aplicabilidad potencial de los resultados para la gestión de la salud pública.

Así, los resultados obtenidos pueden ofrecer importantes subsidios para la práctica clínica al evidenciar los factores de riesgo para COVID prolongada, contribuyendo directamente a la calificación de los servicios de salud - especialmente la APS y la vigilancia de la salud - a través de la formulación de protocolos clínicos dirigidos a la detección temprana de síntomas y la estratificación del riesgo en función de variables sociodemográficas y clínicas. Tales medidas contribuyen a una atención más resolutiva de los problemas, al fortalecimiento de la vigilancia y a un mejor uso de los recursos disponibles.

En el contexto de la enfermería, los hallazgos refuerzan la importancia del papel estratégico de los profesionales en la vigilancia clínica, la recepción y el seguimiento continuo de los usuarios que tienen síntomas persistentes después de la infección por COVID-19. La evidencia del estudio respalda prácticas como la escucha calificada y la planificación de la atención, lo que permite un enfoque más proactivo e individualizado. El reconocimiento de los síntomas más frecuentes, combinado con la valorización de la notificación subjetiva y la consideración de factores como el sexo, la edad y la vacunación, contribuye a una atención más humanizada y resolutiva de acuerdo con la evidencia científica.

A pesar de las contribuciones relevantes, este estudio tiene algunas limitaciones que deben considerarse en la interpretación de los resultados. La principal limitación se refiere a la dependencia de datos secundarios de registros médicos electrónicos, que pueden contener registros incompletos o inconsistentes. Esta limitación puede haber llevado a un subregistro de síntomas o a la omisión de información clínica relevante, comprometiendo la precisión en la identificación de casos de COVID prolongada. Otra limitación de la encuesta se refiere al tamaño de la muestra, que no capta la diversidad de la población brasileña. Además, el diseño transversal adoptado no permite establecer relaciones causales entre los factores analizados y el desarrollo de la COVID persistente, restringiendo el análisis a asociaciones estadísticas.

El uso de información de un solo municipio también limita la generalización de los hallazgos a otras regiones, especialmente considerando las desigualdades regionales en el acceso a la salud, la estructura de la APS y la cobertura de vacunación en Brasil. Además, la definición de COVID prolongada utilizada, aunque basada en la propuesta de la OMS, depende de la presencia de síntomas autorreferidos y del retorno del paciente al servicio de salud después de la fase aguda, lo que puede excluir a individuos que, incluso con síntomas persistentes, no buscaron atención.

## Conclusión

Este estudio mostró que las personas mayores tenían una mayor prevalencia de dificultad para respirar; las mujeres tenían una mayor prevalencia de pérdida de cabello como síntoma de COVID prolongada; y los individuos no vacunados tenían una mayor prevalencia de dolor torácico. Determinar los síntomas más prevalentes de COVID prolongada, considerando el grupo de edad, el sexo y el estado de vacunación, puede contribuir a la evaluación clínica de la enfermedad, así como a la identificación de posibles factores de riesgo para algunos síntomas manifestados en la enfermedad, ayudando a desarrollar protocolos que orienten el diagnóstico y el manejo clínico de la enfermedad.

Los hallazgos también subrayan la importancia de la vacunación contra la COVID-19 como medida preventiva no solo contra las formas graves de la enfermedad aguda, sino también contra las manifestaciones prolongadas. En este sentido, las estrategias para ampliar la cobertura de vacunación y reforzar las campañas públicas son esenciales para reducir el impacto del COVID prolongada en la población.

Finalmente, aunque el estudio se realizó en un solo municipio, sus resultados son aplicables a contextos similares, como ciudades medianas con organización de atención comparable. Aun así, se recomienda realizar estudios multicéntricos, métodos longitudinales y la incorporación de variables sociales y de comportamiento, muestras representativas de diferentes regiones del país, con el fin de validar los hallazgos y profundizar el conocimiento sobre los factores asociados a la COVID prolongada en el contexto brasileño.

## Data Availability

Los conjuntos de datos relacionados con este artículo estarán disponibles previa solicitud al autor correspondiente.
